# ITR-Seq, a next-generation sequencing assay, identifies genome-wide DNA editing sites in vivo following adeno-associated viral vector-mediated genome editing

**DOI:** 10.1186/s12864-020-6655-4

**Published:** 2020-03-17

**Authors:** Camilo Breton, Peter M. Clark, Lili Wang, Jenny A. Greig, James M. Wilson

**Affiliations:** Gene Therapy Program, University of Pennsylvania Perelman School of Medicine, 125 South 31st Street, Suite 1200, Philadelphia, PA 19104 USA

**Keywords:** Genome editing, Off-targets, Next-generation sequencing, In vivo, Editing, AAV integration

## Abstract

**Background:**

Identifying nuclease-induced double-stranded breaks in DNA on a genome-wide scale is critical for assessing the safety and efficacy of genome editing therapies. We previously demonstrated that after administering adeno-associated viral (AAV) vector-mediated genome-editing strategies in vivo, vector sequences integrated into the host organism’s genomic DNA at double-stranded breaks. Thus, identifying the genomic location of inserted AAV sequences would enable us to identify DSB events, mainly derived from the nuclease on- and off-target activity.

**Results:**

Here, we developed a next-generation sequencing assay that detects insertions of specific AAV vector sequences called inverted terminal repeats (ITRs). This assay, ITR-Seq, enables us to identify off-target nuclease activity in vivo. Using ITR-Seq, we analyzed liver DNA samples of rhesus macaques treated with AAV vectors expressing a meganuclease. We found dose-dependent off-target activity and reductions in off-target events induced by further meganuclease development. In mice, we identified the genomic locations of ITR integration after treatment with Cas9 nucleases and their corresponding single-guide RNAs.

**Conclusions:**

In sum, ITR-Seq is a powerful method for identifying off-target sequences induced by AAV vector-delivered genome-editing nucleases. ITR-Seq will help us understand the specificity and efficacy of different genome-editing nucleases in animal models and clinical studies. This information can help enhance the safety profile of gene-editing therapies.

## Background

Genome-editing therapies disrupt normal gene function by creating double-stranded breaks (DSBs) in DNA, induced by a nuclease such as CRISPR-Cas9, within a targeted locus. The host cell subsequently repairs these DSBs, thus resulting in edited alleles that can contain insertions and deletions (indels) or more complex genomic rearrangements [1]. The specificity of these nucleases is conferred by either 1) a single-guide RNA (sgRNA) in the case of the CRISPR-Cas system [2]; 2) a rational design and evolution of sequence-specific DNA-binding domains fused to a nuclease (e.g., TALENs or zinc fingers [3]); or 3) engineered versions of restriction enzymes (e.g., meganucleases) [4, 5]. In spite of efforts by the scientific community to increase the on-target efficiency and specificity of these nucleases, they often exhibit off-target editing effects. Assessing the location and frequency of nuclease target sites is a critical step towards evaluating the safety and efficacy of genome-editing therapies.

Researchers have developed a variety of approaches to identify and quantify the on- and off-target activity of genome editing nucleases to better understand the elements that govern nuclease specificity and to improve the safety profile of these therapies [6]. Nuclease specificity and activity can be studied by first identifying the DSB, either in cultured cells or in animal models, created as a consequence of their nuclease activity [7, 8]. Approaches for determining nuclease specificity include cell-free methods, such as Site-Seq [9], Digenome-seq [10], and Circle-Seq [11], and in vitro methods, such as GUIDE-Seq [12] and Integrative-Deficient Lentiviral Vectors Capture [13, 14]. In addition, during the preparation of this manuscript, Hanlon et al. published an assay, similar to ours, to identify AAV integration after CRISPR-Cas9-mediated editing to identify on- and off-target sites [15].

One of the currently preferred methods for characterizing the off-target activity of nucleases is GUIDE-Seq because 1) it requires a minimal number of components; 2) software is readily available to identify off-target sites; and 3) it can detect low-abundance off-target sites. However, these in vitro analyses might not accurately predict the number and rate of off-target activity in vivo, as the differences in nuclease intracellular levels and the chromatin state between these two conditions (in vitro and in vivo) might affect the editing activity within the on- and off-target sites [16].

Assays such as Breaks Labeling In Situ and Sequencing (BLISS) [17] or End-Seq [18] require an initial cell-fixation step to preserve the free DSB ends. The BLISS approach captures these ends by ligating adapters containing a T7 promoter. An in vitro transcription assay then amplifies the adjacent DNA, producing RNA to construct Illumina libraries for next-generation sequencing (NGS) analysis [17]. The End-Seq method captures DSB ends by ligating biotinylated hairpin adapters [18]. NGS analysis of BLISS and End-Seq NGS libraries makes it possible to accurately determine the sites of nuclease-induced DSBs by selecting sites with homology to the intended target sequence. However, this analysis is only capable of identifying DSBs present at a discreet time point, given that the adapters can only be ligated to free DSB ends. Therefore, these assays cannot capture repaired DSBs created at earlier time points.

To accelerate the translation of gene-editing platform technologies into the clinic, we need to accurately quantify the location and frequency of nuclease-induced DSBs in vivo. Our current strategy to edit endogenous genes within the liver involves intravenous (IV) administration of a gene therapy vector expressing a nuclease. By using adeno-associated virus (AAV) serotype 8 (AAV8), we can effectively target hepatocytes and induce nuclease-mediated genome editing. However, due to the creation of DSBs following genome editing, there is the potential of integrating a fragment of the vector sequences such as inverted terminal repeat (ITR) sequences [19–27]. Therefore, determining the genomic location of inserted ITR sequences would enable us to characterize on- and off-target DSB events.

Here, we developed an assay called ITR-Seq to determine the number and genomic location of these integration events in vivo. We designed an ITR sequence-specific primer to amplify the ITR-genomic DNA junction that occurs after the integration of ITRs directly from primary tissue. We used ITR-Seq to identify and rank genome-wide target sites for five independent nucleases in vivo. Our results indicate that ITR-Seq is a highly sensitive and specific assay for in vivo sites of DSBs and significantly advances our ability to assess the safety and efficacy of translational genome-editing therapies.

## Results

### Developing the ITR-Seq assay to evaluate meganuclease activity in non-human primates

We sought to develop a methodology for unbiased, genome-wide identification of sites of ITR integration. Using the AAV ITR as a tag for identifying DSBs, we can measure the off-target activity of genome-editing nucleases in vivo. Our method is based on previous research that demonstrated AAV ITR sequence integration into the host’s genomic DNA after the occurrence of DSBs [21–25, 27–31].

**Fig. 1:**
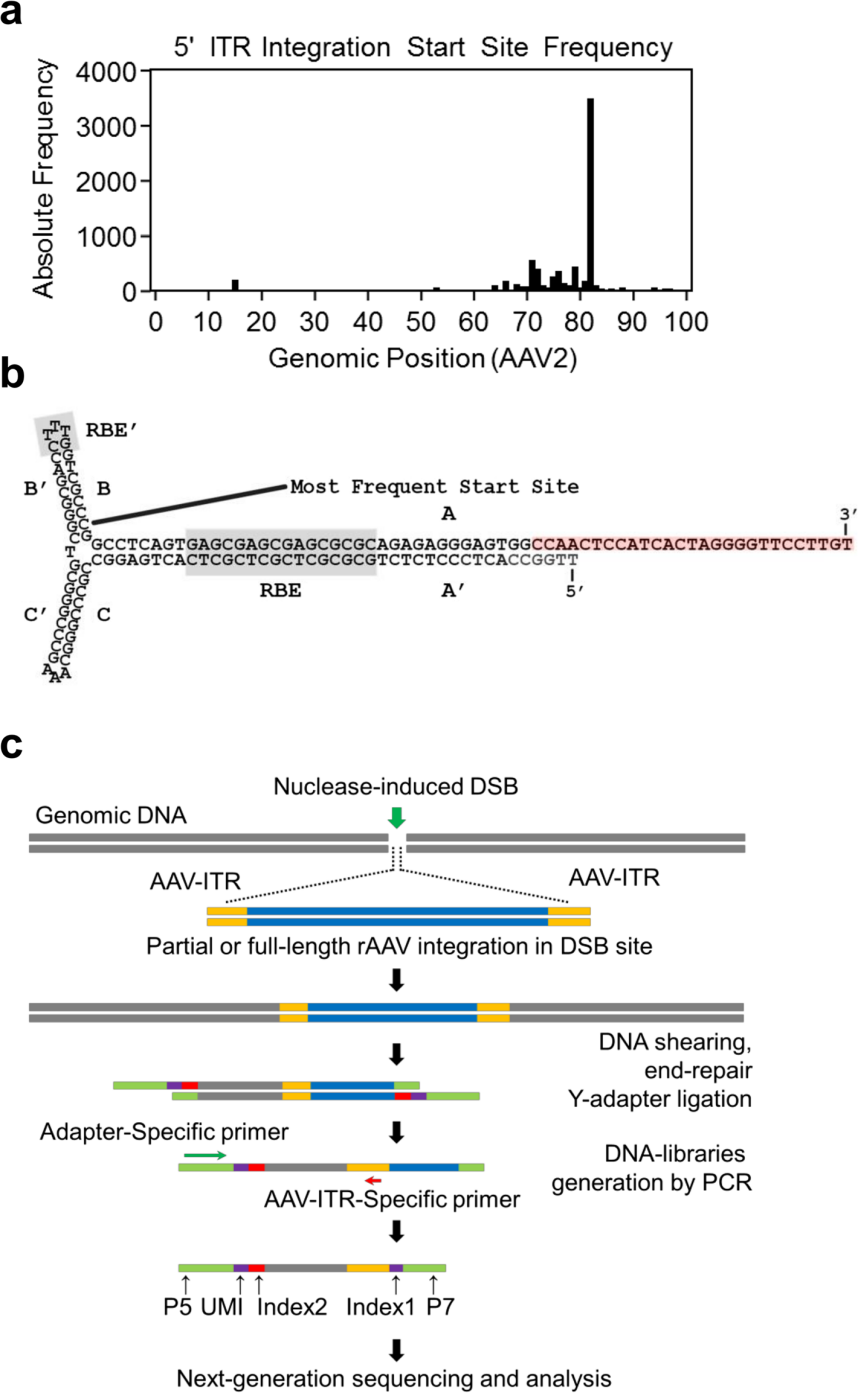
Sequence analysis of AAV ITRs integrated into genomic DNA. **a.** Meta-analysis of on-target AMP-Seq data for all AAV8-M1PCSK9- and AAV8-M2PCSK9-treated liver samples (SRR6343442). Our goal was to identify the most frequent ITR integration start site within the vector ITR. **b.** Secondary structure of the AAV2 5′ ITR (NC_001401.2). The most frequently integrated start site position is shown. The ITR-Seq primer (GSP_ITR3.AAV2) binding site is highlighted in red. A-A’, B-B′, and C-C′, palindromic arms; RBE, Rep-binding element; TRS, terminal resolution site. **c.** Schematic diagram of the ITR-Seq protocol used for genome-wide identification of ITR integration sites

We reanalyzed the NGS reads that were generated in a previous study where we characterized the on-target regions following administration of AAV vectors expressing two generations of a meganuclease (AAV8-M1PCSK9 and AAV8-M2PCSK9) [26]. Our aim was to identify the most common AAV ITR sequences that were integrated into the meganuclease on-target loci in the PCSK9 gene. Based on a peak in absolute frequency at position 82 of the AAV2 reference genome (Fig. 1a), we determined that the most frequent base position of ITR integration occurs 5′ upstream of the Rep-binding element. We used this information to design an ITR-specific primer that hybridizes 5′ upstream of the observed ITR-integration start site (shown in red in Fig. 1b). We used this primer in a novel NGS assay, based on anchored multiplexed PCR, to identify the ITR-genomic DNA junction following insertional mutagenesis (Fig. 1c). We named this method ITR-Seq.

To use the ITR-Seq assay for sample analysis, we first isolated DNA from the tissues of animals treated with nuclease-expressing AAV vectors. We sheared the DNA and ligated it to Y-adapters, as described in previous reports [12]. Following two rounds of PCR using the ITR-specific primer described above and adapter-specific primers, we produced NGS-compatible libraries. After sequencing, we computationally identified the resulting amplicons that contain both the amplified ITR sequence and adjacent genomic DNA sequences. We also determined the location and frequency of genome-wide ITR integration sites. By requiring that the ITR integrates in both the forward and reverse strand orientations, we aimed to further reduce the number of false positives and identify high-confidence ITR-integration sites. For each sample, we produced a rank-ordered list of nuclease target sites (ITR-Seq rank) and sorted the sites in decreasing order by the total number of observed ITR-integration events per locus (ITR-Seq reads). We generated ITR-Seq reports at the end of the computational analysis (Dataset S1 and S2) with the most probable off-target sequence (based on homology to the intended target sequence), the genomic location, and the ITR-Seq rank (according to the number of NGS reads mapping to the corresponding locus).

### ITR-Seq identifies nuclease off-target sites in rhesus macaques

**Fig. 2:**
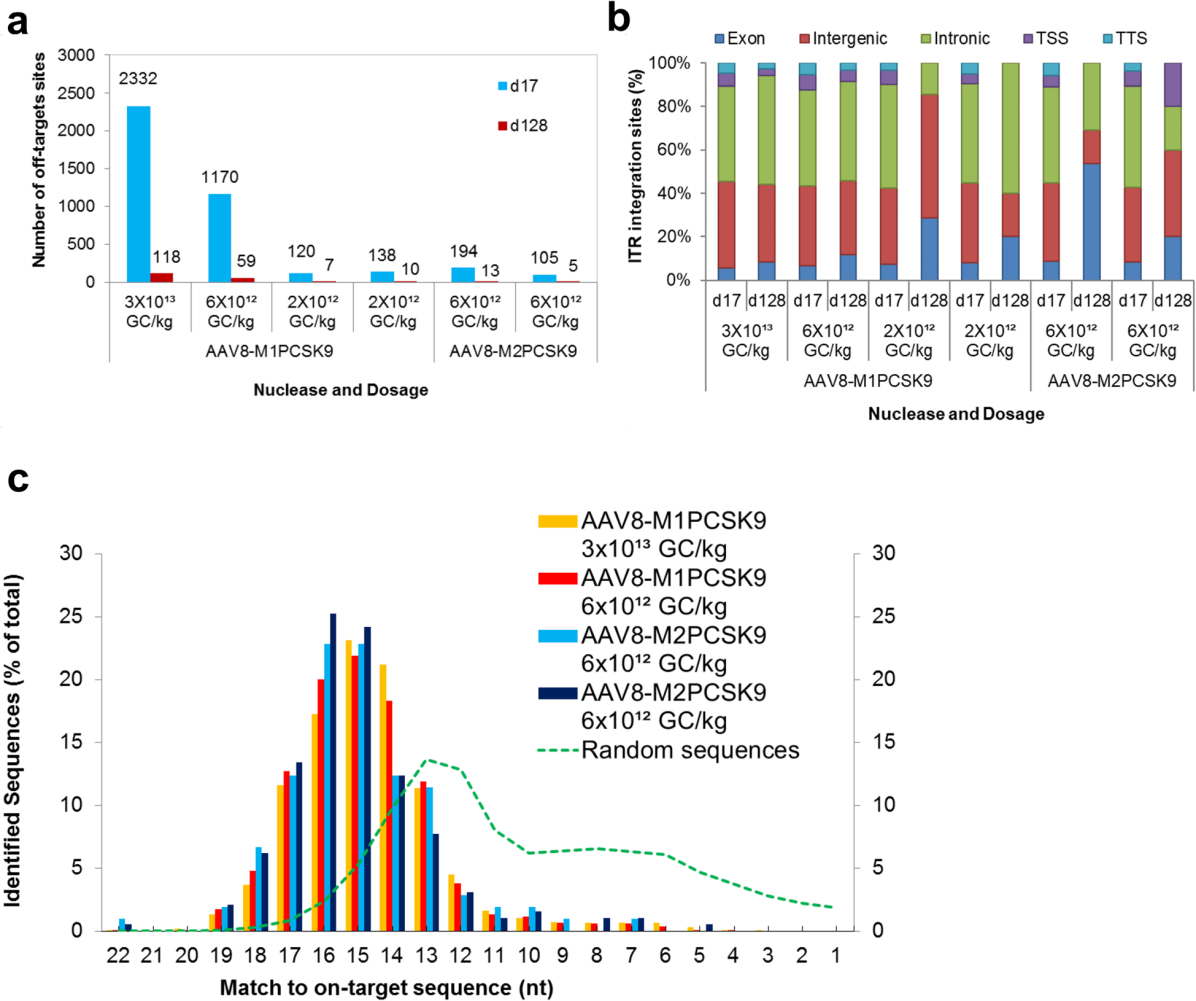
Analyzing on- and off-target activity of AAV8-M1PCSK9 and AAV8-M2PCSK9 in vivo. **a.** ITR-Seq-identified integration sites in liver samples treated with AAV8-M1PCSK9 and AAV8-M2PCSK9. Samples were collected on day 17 and 128 following vector administration. **b.** Functional annotation of ITR-identified integration sites. Here, we show the number of sites within exons, introns, intergenic regions, transcription start sites (TSS), and transcription termination sites (TTS). **c.** Distribution of ITR-integration sites on days 17/18 for two animals treated with either M1PCSK9 or M2PCSK9 (colored bars). Computationally generated random DNA sequences are represented by the green dotted line and are based on the number of nucleotides that match the intended target sequence (represented as a percent of all identified sites)

We next used the ITR-Seq assay to further analyze the on- and off-target effects of meganucleases in rhesus macaques that had previously been administered AAV8-M1PCSK9 or AAV8-M2PCSK9 (Fig. 2). As previously described, rhesus macaques received either one of three doses of AAV8-M1PCSK9 [3 × 10^13^ genome copies (GC)/kg, 6 × 10^12^ GC/kg, or 2 × 10^12^ GC/kg] or a single dose of AAV8-M2PCSK9 (6 × 10^12^ GC/kg) [26]. We took a liver biopsy of each macaque on days 17 and 128 post-vector administration to evaluate on- and off-target editing. Across all samples from meganuclease-treated macaques, the top ITR-Seq rank was the on-target locus (PCSK9 target site sequence TGGACCTCTTTGCCCCAGGGGA, chr1:54708864–54,708,885) [26]. For both generations of the meganuclease (i.e., AAV8-M1PCSK9 and AAV8-M2PCSK9), the number off-target sites identified by ITR-Seq depended on both the administered vector dose and the sample time point. The number of off-target sites decreased as a function of time (e.g., day 17 had more off-target sites than day 128; see Fig. 2a). We observed a dose-dependent effect among the non-human primates treated with AAV-M1PCSK9, where the highest AAV-M1PCSK9 dose (3 × 10^13^ GC/kg) resulted in the highest number of off-target sites (2332 off-target sites at d17) while those administered with the lowest tested dose (2 × 10^12^ GC/kg) resulted in the lowest number of off-target sites for this group (120 and 138 off-target sites at d17, Fig. 2a). Animals that received the second-generation engineered meganuclease M2PCSK9 had fewer identified off-target sites than animals treated with the first-generation meganuclease M1PCSK9 at the same dose. In total, we observed 1170 different off-target sites after administering AAV8-M1PCSK9 at a dose of 6 × 10^12^ GC/kg. By contrast we only observed 194 and 105 off-target sites in the two macaques that received AAV8-M2PCSK9 at the same dose (Fig. 2a).

We then assessed the frequency of AAV integration in the on- and off-target sites by dividing the total number of ITR-Seq reads in the on- and off-target regions by the total number of ITR-Seq reads (on- and off-target). The distribution of ITR-Seq reads for each treated non-human primate at day 17 and d128 is shown in Figure S1. Interestingly, we observed an increase in the on-target % from d17 to d128, indicating that by day 128 between 60 and 90% of the AAV-integrated sequences are in the on-target region (Figure S1). This AAV integration frequency was significantly different (Wilcoxon singed-rank test) between the on- and off-target regions (*p* = 0.03125) at day 128 post AAV administration.

We randomly selected a subset of ITR-Seq-identified off-target sites from the results of day 17 liver samples from macaques treated with 3 × 10^13^ or 6 × 10^12^ GC/kg of AAV8-M1PCSK9. We then investigated the presence of ITR sequences in these loci by AMP-Seq, as previously described [26, 32], using gene-specific primers flanking the identified off-target sequences (Additional File 1: Table S1). We found reads containing ITR sequences in 24 (for the 3 × 10^13^ GC/kg dose) or 21 (for the 6 × 10^12^ GC/kg dose) out of 27 interrogated loci, with the highest percentage of ITR integration (ITR-containing reads) corresponding to those off-target sites with high ITR-Seq rank (Additional File 1: Table S1). Importantly, in both animals we found the highest level of editing in those loci with the highest ITR-Seq rank, suggesting a correlation between the ITR-Seq rank, the percentage of ITR integration, and the indel percentage. For some of these loci, we could not detect ITR sequences integrated by the AMP-Seq assay (Additional File 1: Table S1); this could be due to a lower sensitivity of the AMP-Seq assay to detect ITR integration, as the ITR-Seq method uses the actual ITR sequences as a starting point for the amplification. Similarly, we were able to observe ITR sequences in a couple of sites not shown in the ITR-Seq results (e.g., 20:359062–359,285 and 7:165269225–165,269,449 for liver samples of animals treated with 3 × 10^13^ GC/kg of AAV8-M1PCSK9; Dataset S1). However, we found these sites with the ITR-Seq method in animals treated with 6 × 10^12^ GC/kg of AAV8-M1PCSK9 (Dataset S1), suggesting that our current protocol does not capture 100% of the ITR integration sites. Therefore, the sensitivity of the ITR-Seq method can be improved.

We then annotated the identified sites of ITR integration based on the function of the DNA region (Fig. 2b). Regardless of the number of identified nuclease target sites, the genomic distribution of target sites (intergenic, intronic, or exonic regions) was reproducible among meganuclease-administered macaques. Generally, most target sites reside within introns, followed by intergenic regions of the genome (Fig. 2b). The meganucleases we evaluated here have a 22-nucleotide target site within the PCSK9 gene. Therefore, we evaluated the number of conserved nucleotides between the target DNA sequence and the DNA sequence of each off-target site (Fig. 2c). The distribution of matches to the on-target sequence appeared to follow a Gaussian distribution with a mean of 15–16 nucleotides. This indicates that the majority of ITR-Seq-identified off-target sites have six or seven mismatches between the targeted DNA sequence motif and the genomic DNA sequence of each target site (Fig. 2c). To assess if this level of homology resulted from editing in sequences similar to the meganuclease target sequence or simply due to chance, we generated ten million random DNA regions (40 bp in length) within the rhesus macaque genome. We then attempted to identify the sequence that was most similar to the meganuclease target sites using the same algorithm from the ITR-Seq protocol (Fig. 2c, green line). Unlike ITR-Seq-identified sequences, random sequences share an average of 11–12 mismatches with the intended target sequence. This indicates that the ITR is mostly integrated in the targets with a certain degree of homology to the intended target sequence. We then analyzed a subset of these off-target sequences to evaluate the distribution of mismatches between the on-target sites and a subset of the top-ranked off-target sites. We found that these mismatches were more likely to occur in particular nucleotide positions (nucleotides 1, 4, 12, 13, 14, and 21; Figure S2), while some nucleotides remained unchanged between the on- and off-target sites (nucleotides 2, 3, 5, 11, 15, 16, and 18; Figure S2).

### Comparing GUIDE-Seq and ITR-Seq identification of off-target sites

**Fig. 3:**
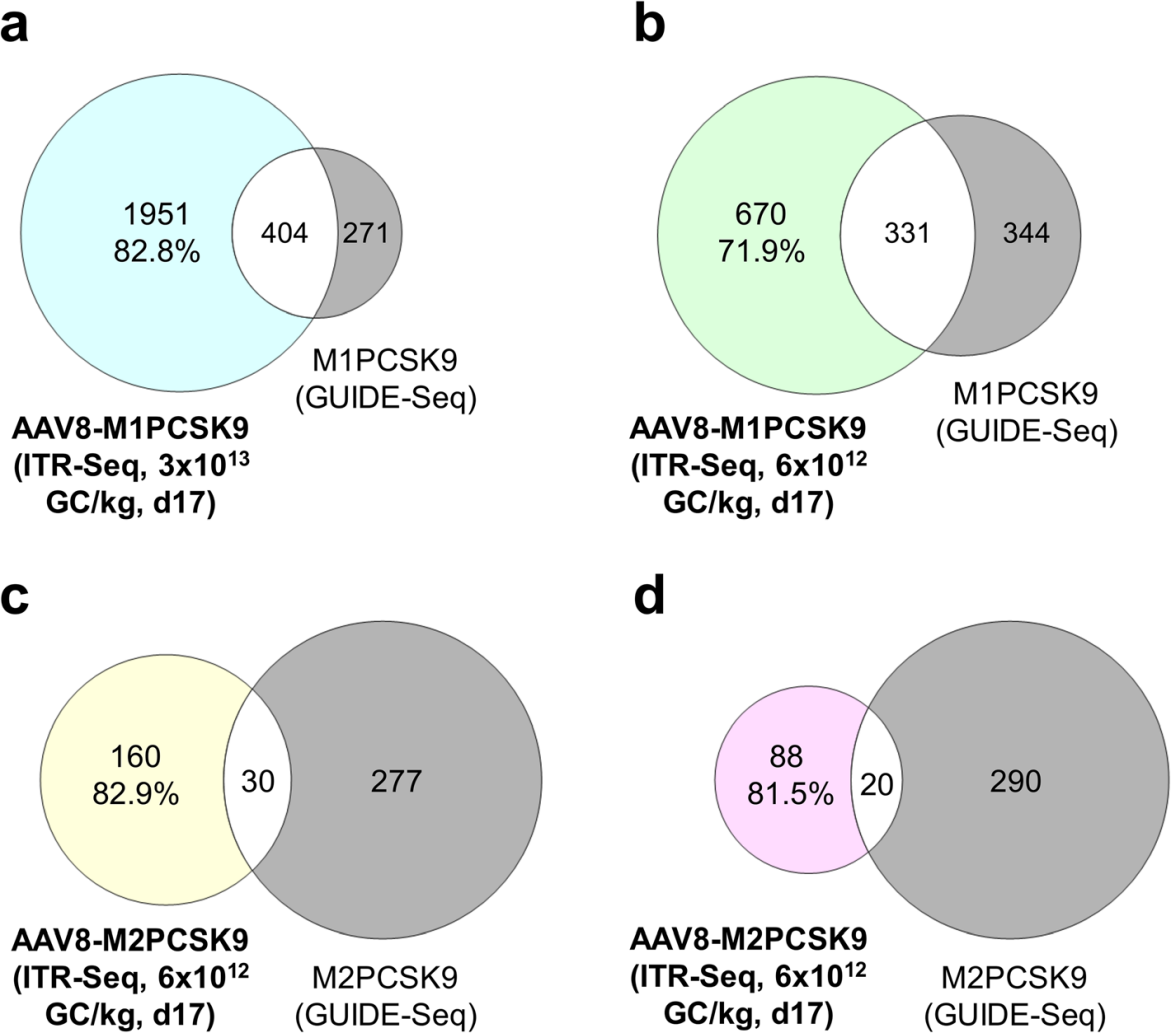
Comparing GUIDE-Seq and ITR-Seq in terms of off-target identification. Sample set intersections of identified target sites obtained from in vivo ITR-Seq (coloured) from two doses of AAV8-M1PCSK9 (3 × 10^13^ GC/kg; panel **a**; and 6 × 10^12^ GC/kg; panel **b**) or one dose of AAV8-M2PCSK9 (6 × 10^12^ GC/kg; panels **c** and **d**). We obtained target sites on day 17 post-AAV administration. In vitro GUIDE-Seq for M1PCSK9 or M2PCSK9 is shown in gray. Off-target sites identified by ITR-Seq but not by GUIDE-Seq (coloured sections) are indicated as a percent of the total number of off-target sites that were identified by in vivo ITR-Seq. White sections of the Venn diagrams show the proportion of off-target sites that were identified by both ITR-Seq and GUIDE-Seq

For each meganuclease (M1PCSK9 and M2PCSK9), we had previously performed GUIDE-Seq analyses on LLC-MK2 cells transfected with plasmids to express the nucleases in order to identify off-target sites in vitro [26]. We selected the M1PCSK9 and M2PCSK9 off-target locations from the list of GUIDE-Seq-identified off-target sites in vitro. Using rhesus macaques, we then validated these predicted off-target sites in vivo using amplicon sequencing of off-target loci [26]. Both amplicon sequencing [26] and ITR-Seq (Fig. 2a) exhibited a dose- and time-dependent reduction in off-target editing efficiency. We compared these previous results with our evaluation of off-target sites in vivo using ITR-Seq (Fig. 3). By identifying off-target sites with no homology to the indented target sequence, we found approximately the same number of off-target sites across two independent experiments using either M1PCSK9 (1093 and 1499, for GUIDE-Seq experiment 1 and 2, respectively) or M2PCSK9 (568 and 651, for GUIDE-Seq experiment 1 and 2, respectively). We compared sites that were identified by both GUIDE-Seq in vitro experiments to the off-target sites identified by ITR-Seq. For this study in rhesus macaques, we performed ITR-Seq on DNA samples from liver biopsies taken on day 17 post-nuclease administration. We compared the GUIDE-Seq- and ITR-Seq-identified off-target sites in macaques that received a dose of 3 × 10^13^ GC/kg of AAV8-M1PCSK9 (Fig. 3a), 6 × 10^12^ GC/kg of AAV8-M1PCSK9 (Fig. 3b), or 6 × 10^12^ GC/kg of AAV8-M2PCSK9 (two animals; Fig. 3c, d). Most (71.9–82.9%) off-target sites were identified exclusively by ITR-Seq, and not by GUIDE-Seq (see colored sections of Fig. 3). Interestingly, among animals that received the second-generation nuclease (M2PCSK9), both ITR-Seq and GUIDE-Seq identified fewer off-target sites (see white sections of Fig. 3).

We have validated a set of GUIDE-Seq-identified high- and low-rank off-target sites, which were ranked according to the number of reads [26]. We did this by quantifying the indel percentage, which we determined using amplicon sequencing of off-target loci in samples taken from macaques administered with meganucleases on days 17/18 and 128/129 [26]. We counted off-target sites that exhibited a significantly higher indel percentage than untreated peripheral blood mononuclear cell (PBMC) DNA control samples as positive (bold typeface in Additional File 1: Table S2).

Here, we evaluated whether ITR-Seq can identify positive GUIDE-Seq off-target sites. Given that we re-analyzed the same DNA used to validate the GUIDE-Seq off-target sites, we were able to assess the sensitivity of our method in detecting the positive off-target loci. Among macaques administered with 3 × 10^13^ and 6 × 10^12^ GC/kg of AAV8-M1PCSK9, ITR-Seq only failed to identify two high-rank and two low-rank positive GUIDE-Seq off-target sites (S3 Table). Among macaques administered with AAV8-M2PCSK9, ITR-Seq correctly identified most of the positive off-target sites and only missed three low-rank positive off-target sites in one animal and three high-rank positive off-target sites in the other macaque. Taken together, these results clearly indicate that ITR-Seq and not GUIDE-Seq could identify the vast majority of in vivo off-target sites. This suggests that unlike amplicon sequencing of GUIDE-Seq-predicted off-target sites, ITR-Seq more accurately examines the activity of AAV-delivered nucleases in vivo.

### Evaluating ITR-Seq assay as a tool for identifying guide RNA-dependent nuclease off-target sites in mice

We tested whether ITR-Seq can detect the on- and off-target activity of a variety of guide RNA-dependent nucleases (i.e., SaCas9, LbCpf1, and AsCpf1) that are commonly used in preclinical studies. We also evaluated whether ITR-Seq analysis is compatible with the distinct types of DSB ends created by these nucleases (blunt for SaCas9 and 5′ overhang for Cpf1).

We co-administered newborn C57BL6/J mice with AAV vectors expressing SaCas9, LbCpf1, or AsCpf1 nucleases at a dose of 3 × 10^11^ GC/mouse together with vectors expressing the corresponding guide RNAs at a dose of 2 × 10^12^ GC/mouse (sgRNA, Table 1). We sacrificed mice on day 21 post-vector administration, harvested the liver, and extracted the DNA for ITR-Seq analysis in order to evaluate the frequency and location of nuclease-mediated DNA cleavage sites. We co-administered additional groups of newborn mice with vectors expressing SaCas9 or LbCpf1 as above at a dose of 10^11^ or 3 × 10^11^ GC/mouse. The second vector expressed sgRNA and the human coagulation factor IX (hFIX) transgene (instead of the donor DNA sequence used in the first experiment) at a dose of 10^12^ GC/mouse. Our goal was to evaluate the effect of vector dose on ITR integration. We sacrificed these mice on day 70 post-vector administration and subjected DNA samples from the liver to ITR-Seq analysis (Table 1).

**Table 1:** Using ITR-Seq for on- and off-target evaluation of sgRNA-dependent nucleases

Vector 1 (GC/kg dose)	Vector 2 (GC/kg dose)	Time point(days)	Indel% (on-target)		Off-Target Sites		Target locus	Target Sequence
			Mouse A	Mouse B	Mouse A	Mouse B		
AAV8-SaCas9 (3 × 10^11^)	AAV8-sgRNA1 (2 × 10^12^)	21	28.05	32.59	3	4	ASS1 (chr2:31518639–31518658)	ACAGGACTCCCAGAGTTAGA
AAV8-LbCpf1 (3 × 10^11^)	AAV8-sgRNA1 (2 × 10^12^)	21	14.35	23.87	1	2	ASS1 (chr2:31518480–31518502)	CAAATGGCAGGAAGAATTCACGG
AAV8-LbCpf1 (3 × 10^11^)	AAV8-sgRNA2 (2 × 10^12^)	21	33.42	27.64	4	3	ASS1 (chr2:31519012–31519034)	TGGCTGGAAATATTAGGGCAACT
AAV8-AsCpf1 (3 × 10^11^)	AAV8-sgRNA1 (2 × 10^12^)	21	1.22	0.38	3	1	ASS1 (chr2:31518480–31518502)	CAAATGGCAGGAAGAATTCACGG
AAV8-AsCpf1 (3 × 10^11^)	AAV8-sgRNA2 (2 × 10^12^)	21	0.28	0.12	2	1	ASS1 (chr2:31519012–31519034)	TGGCTGGAAATATTAGGGCAACT
AAV8-SaCas9 (3 × 10^11^)	AAV8-sgRNA1 (1 × 10^12^)	70	26.94	29.11	10	12	ASS1 (chr2:31518639–31518658)	ACAGGACTCCCAGAGTTAGA
AAV8-SaCas9 (1 × 10^11^)	AAV8-sgRNA1 (1 × 10^12^)	70	21.39	25.98	7	9	ASS1 (chr2:31518639–31518658)	ACAGGACTCCCAGAGTTAGA
AAV8-LbCpf1 (1 × 10^11^)	AAV8-sgRNA2 (1 × 10^12^)	70	4.91	4.90	5	6	ASS1 (chr2:31519012–31519034)	TGGCTGGAAATATTAGGGCAACT
AAV8-LbCpf1 (3 × 10^11^)	AAV8-sgRNA2 (1 × 10^12^)	70	7.69	8.48	10	4	ASS1 (chr2:31519012–31519034)	TGGCTGGAAATATTAGGGCAACT

We administered AAV8 expressing the indicated nucleases to mice at the indicated doses. This table shows the targeted gene and genomic position, on-target indel percentage, and the number of ITR-Seq-identified off-target sites.

Aside from one animal treated with AsCpf1-sgRNA2, the on-target locus (mASS1) was the target with the highest number of reads in ITR-Seq analysis. In addition, all of the evaluated sgRNA-directed nucleases exhibited high specificity for the targeted locus with an on-target indel percentage of up to 33% (Table 1). Importantly, mice treated with AAV8-SaCas9 exhibited the highest frequency of on-target ITR integration events across all treated samples (Table 1). Despite showing comparable on-target editing, we observed lower on-target ITR integration events in livers treated with AAV8-LbCpf1 versus AAV8-SaCas9 (S3 Table). AAV8-AsCpf1 had very low editing efficiency at the on-target locus with a maximum indel percentage of 1.22%, as assessed by targeted amplicon sequencing (Table 1).

All of the ITR-Seq-identified off-target sites for the CRISPR nucleases resided within annotated mouse genes, with the most common site being the on-target locus (S3 Table). We identified some low-frequency off-target sites for the tested sgRNAs, most of which had low homology to the target sequence. One off-target site that we identified for the AAV8-SaCas9 sgRNA was located within the locus of a known oncogene, NOTCH2 (S3 Table). Importantly, we found this off-target site in mice administered with AAV8-SaCas9 at a dose of 3 × 10^11^ GC/mouse and AAV-sgRNA1 at a dose of 2 × 10^12^ GC/mouse. However, this site was absent following administration of 10^12^ GC/mouse of AAV-sgRNA, a twofold lower dose. Thus, only a higher dose of AAV8-sgRNA1 led to editing at this low abundance off-target site (Table 1 and S3 Table).

### Analyzing nuclease-independent events in mice and non-human primates

Among mice injected with AAV8 expressing a CRISPR-related nuclease and an sgRNA, ITR integration occurred in the loci targeted by the sgRNA. Interestingly, ITR integration also occurred in a seemingly sgRNA-independent fashion, as we observed AAV ITR integration in control samples where there was no functional sgRNA (Table 2) [33, 34]. To evaluate the frequency and scope of nuclease-independent ITR integration in non-human primates, we evaluated the liver DNA of rhesus macaques administered with AAV-eGFP at a dose of 3 × 10^12^ GC/kg (Table 2). We detected nuclease-independent integration events, although not in identified genes, as positive ITR insertions. Importantly, our method appears to exclusively identify integrated ITR sequences as a result of in vivo events. We came to this conclusion based on our finding that we did not identify off-target sites in DNA isolated from PBMCs of untreated rhesus macaques spiked with AAV.eGFP DNA (data not shown).

**Table 2:** Annotation of nuclease-independent, ITR-Seq-identified off-target sites to genomic location

Sample		Genomic location	ITR-Seq reads	Gene Symbol
AAV8-SaCas9 + AAV8-sgRNA-ctrl	Mouse A	2:98666941–98666946	5	Gm10800
AAV8-SaCas9 + AAV8-sgRNA-ctrl	Mouse B	5:90465978–90465995	8	Alb
AAV8-LbCpf1 + AAV8-sgRNA-ctrl	Mouse A	–	–	–
AAV8-LbCpf1 + AAV8-sgRNA-ctrl	Mouse B	2:98667196–98667216	29	Gm10800
		2:31519035–31519051	22	Ass1
		5:90474802–90474819	3	Alb
AAV8-AsCpf1 + AAV8-sgRNA-ctrl	Mouse A	–	–	–
AAV8-AsCpf1 + AAV8-sgRNA-ctrl	Mouse B	2:31518461–31518481	106	Ass1
		5:90472660–90472675	7	Alb
		9:103219199–103219216	3	Trf
AAV8-SaCas9 + AAV8-sgRNA-ctrl	Mouse A	–	–	–
AAV8-SaCas9 + AAV8-sgRNA-ctrl	Mouse B	2:98666940–98666966	67	Gm10800
		5:90476386–90476411	12	Alb
		5:90472060–90472079	11	Alb
		9:21837574–21837595	10	Dock6
		9:69244457–69244483	5	Rora
		5:90505454–90505480	4	Alb
AAV8-LbCpf1 + AAV8-sgRNA-ctrl	Mouse A	–	–	–
AAV8-LbCpf1 + AAV8-sgRNA-ctrl	Mouse B	–	–	–
AAV8.EGFP (rhesus macaque)	d7 - 1w	6:157029767–157029776	12	–
AAV8.EGFP (rhesus macaque)	d35 - 1w	2:7161434–7161476	104	
AAV8.EGFP (rhesus macaque)	d45 - 1 m	–	–	–

This table shows a rank-ordered list of identified nuclease off-target sites and the corresponding genome location. For AAV8.EGFP treatments, we injected macaques with AAV at day 7, 35, or 45 (d7, d35, or d45, respectively), and then euthanized them at either one week or one-month post vector administration (1w or 1 m).

## Discussion

Developing an NGS-based assay that can identify and rank nuclease-induced DSBs after in vivo gene editing would significantly advance our ability to evaluate the safety and efficacy of genome editing therapies for translation to human clinical trials. Here, we show that our novel ITR-Seq assay can assess the specificity of any AAV-expressed nuclease in vivo. This method is more sensitive in detecting ITR integration than other NGS-based methods such as the combined approach of GUIDE-Seq and subsequent amplicon sequencing.

In contrast to GUIDE-Seq, ITR-Seq is not a tool for predicting in vivo off-target sites. Rather, ITR-Seq identifies novel sites in the genome where on- and off-target nuclease activity occurred. Indeed, this method identifies AAV ITR integration sites directly from the DNA samples of animals treated with nuclease-expressing AAV. Identified off-target sites can be further analyzed using amplicon sequencing to 1) accurately determine the percent of editing and ITR integration; and 2) obtain a detailed panorama of the nuclease activity in clinically relevant doses and animal models.

Analysis of non-human primate samples by ITR-Seq showed a clear decrease in the number of off-target sites, as well as the total number of ITR-Seq reads for the off-target sequences from d17 to d128 (Fig. 2a). Considering that we observed a relationship between the total AAV dose and the total number of off-target sites (Fig. 2a), we hypothesize that high intracellular expression of the nuclease, as a consequence of the high AAV dose, results in a high number of off-target sites. If these cells are later removed either by the immune system or due to toxicity related to the off-target activity, then a decrease in the total number of off-target sites is expected. More experiments are needed to correctly identify the cause of this off-target reduction over time.

In our studies in mice, the most common nuclease-independent ITR integration events occurred in the Gm10800 and albumin genes. ITR integration in the albumin gene concurs with previous reports that show that the albumin gene is quite susceptible to AAV integration [33]. Furthermore, AAV integration has been reported for genes that are transcriptionally active in the liver [34]. Our findings suggest that the rate of AAV ITR integration can be influenced by 1) the homology between the target sequence; or 2) the blunt or overhang nature of the DNA ends as a result of the nuclease cuts. Detailed studies in the future will be needed to fully elucidate the dynamics of ITR integration.

Alternative methods such as BLISS [17], BLESS [35], and End-Seq [18] can identify sites of DSBs by capturing the DSB ends created by the activity of the nuclease. These methods can accurately identify nuclease-induced DSBs in vivo. However, these methods have the limitation that they can only capture DSBs present at a single time point. This limitation can be partially overcome by analyzing DSBs at multiple time points. Non-restrictive linear-amplification mediated PCR (nrLAM-PCR) coupled with NGS [36], a method similar to ITR-Seq, can detect AAV-ITR integration sites. Researchers have used this method to identify the integration sites of the AAV1-LPLS447X vector, which was developed for treating lipoprotein lipase deficiency in mouse and human DNA samples [29]. Although in theory nrLAM-PCR can detect the off-target activity of nucleases, researchers have not yet directly compared ITR-Seq and nrLAM-PCR to understand the advantages and limitations of these two techniques.

## Conclusions

In conclusion, given that the only requirement of ITR-Seq is using an AAV as a delivery vector, this method can be used to measure the specificity of the nucleases in virtually any organism with an annotated reference genome. Researchers could use ITR-Seq as a companion diagnostic in pre-clinical and clinical studies to evaluate nuclease target sites in longitudinal animal studies that have varying dosages and/or administration routes. This technique can yield invaluable insights into the safety and efficacy of gene editing therapies and ultimately better inform the design of future gene editing therapies. This same approach can evaluate AAV integration events in traditional AAV gene therapy studies. Although the risks of insertional mutagenesis from AAV gene therapy is considered low enough [19, 37] to justify its use for treating rare disabling and lethal diseases, these potential risks may be more relevant as the field evolves to treating less severe acquired diseases.

## Methods

### Animal studies

All animal procedures were performed in accordance with protocols approved by the Institutional Animal Care and Use Committee of the University of Pennsylvania. Mice were obtained from The Jackson Laboratory (Bar Harbor, ME).

### Rhesus macaque studies

DNA samples from previously published studies [26] were used for ITR-Seq analysis. Briefly, AAV8 vectors driving the expression of the meganucleases M1PCSK9 (AAV8.TBG.M1PCSK9.WPRE) or M2PCSK9 (AAV8.TBG.M2PCSK9.WPRE) were administered via a peripheral vein to rhesus macaques (*n* = 4 for M1PCSK9- and *n* = 2 for M2PCSK9-treated animals). Liver biopsies were performed at 17 and 129 days (for AAV8-M1PCSK9) or 18 and 128 days (for AAV8-M2PCSK9) post vector administration [26]. As an untreated control, we used DNA extracted from PBMC samples collected prior to vector administration [26].

To measure nuclease-independent AAV integration events, we analyzed liver DNA samples from a previous published study [38]. Briefly, one-week or one-month-old male rhesus macaques were administered with AAV8.TBG.EGFP at a dose of 3 × 10^12^ GC/kg. Animals were euthanized post-vector administration, and livers were collected.

### Mouse studies

Newborn (0–2 days of age, *n* = 2 per group) C57BL/6 J mice were co-administered by temporal vein injection AAV expressing either SaCas9 (AAV8.TBG.hSaCas9.bGH), LbCpf1 (AAV8.ABP2.TBG-S1.hLbCpf1.bGH), or AsCpf1 (AAV8.ABPS2.TBG-S1.hAsCpf1.PA75) at a dose of 3 × 10^11^ GC/mouse, as well as vectors expressing specific sgRNA (AAV8.U6.sgRNA.mASS1.donor(mASS1)) or untargeted sgRNA as controls (AAV8.U6.sgRNA-ctrl.mASS1.donor(mASS1)) at a dose of 2 × 10^12^ GC/mouse. At 21 days post-vector administration, mice were euthanized by carbon dioxide asphyxiation, death was confirmed by cervical dislocation, and livers were collected.

Additional newborn mice (*n* = 2 per group) were co-administered with vector expressing SaCas9 or LbCpf1 as described above at a dose of 10^11^ or 3 × 10^11^ GC/mouse, with the second vector expressing ASS1-specific-sgRNA and the hFIX transgene (AAV8.U6.sgRNA.mASS1.TBG.hFIX) at a dose of 10^12^ GC/mouse. Livers were collected at 70 days post-vector administration.

### ITR-Seq

The ITR-Seq protocol is a modified version of an anchored PCR reaction [12, 32], in which a single primer is designed to anneal to and amplify outward from the ITR sequence (Fig. 1c). Following ITR integration in the DNA, the primer may be used to amplify the junction of the host genome and the inserted vector ITR sequence (Fig. 1b, c). In order to adequately denature the ordered secondary structure of the integrated ITR, we used a high annealing temperature of 69 °C and designed longer adapter-specific primers (S4 Table).

Amplicons were generated from purified genomic DNA isolated from liver tissue samples. DNA was sheared to an average size of 500 bp using an ME220 focused-ultrasonicator (Covaris, Woburn, MA), purified using AMPure beads (Beckman Coulter, Indianapolis, IN) at a 0.8x ratio, and eluted in 15 μl of elution buffer (Qiagen, Hilden, Germany). End repair was subsequently performed in a total volume of 22.5 μl containing 1 μl of 5 mM dNTP mix (Thermo Fisher Scientific, Waltham, MA), 2.5 μl of 10x SLOW ligation buffer (Enzymatics, Beverly, MA), 2 μl of End-Repair Mix (Low Concentration; Enzymatics, Beverly, MA), 2 μl of 10x buffer for Taq Polymerase (MgCl_2_-free; Invitrogen, Carlsbad, CA), 0.5 μl of non-hot start Taq polymerase (New England BioLabs, Ipswich, MA), 0.5 μl of nuclease-free water (Life Technologies, Waltham, MA), and 14 μl of 400 ng sheared genomic DNA. The mix was incubated at 12 °C for 15 min, 37 °C for 15 min, 72 °C for 15 min, and then held at 4 °C. Unique Y-adapters, with molecular index tags annealed to MiSeq Common Adapters (Illumina, San Diego, CA), were ligated to the end-repaired DNA in the following mix: 1 μl of 10 μM annealed A01-A16 Y-adapter, 2 μl of T4 DNA ligase (Enzymatics, Beverly, MA), and 22.5 μl of the previous end-repaired DNA. The ligation program was 16 °C for 30 min, 22 °C for 30 min, and then held at 4 °C. DNA was then purified by AMPure beads (Beckman Coulter, Indianapolis, IN) at a 0.7x ratio. End-repaired Y-adapter-ligated DNA fragments were amplified by PCR using an ITR-specific primer and an adapter-specific primer (A01-A16_P5_FWD primer) in the following mix (amounts per sample): 11.9 μl of nuclease-free water, 3 μl of 10x buffer for Taq Polymerase (MgCl_2_-free, Invitrogen, Carlsbad, CA), 0.6 μl of 10 mM dNTP mix (Thermo Fisher Scientific, Waltham, MA), 1.2 μl of 50 mM MgCl_2_ (Invitrogen, Carlsbad, CA), 0.3 μl of 5 U/μl Platinum Taq polymerase (Invitrogen, Carlsbad, CA), 1 μl of 10 μM GSP_ITR3.AAV2 primer, 1.5 μl of 0.5 M TMAC (Sigma-Aldrich, St. Louis, MO), 0.5 μl of 10 μM A01-A16_P5_FWD primer with the primer number matching the adapter number (e.g., A01_P5_FWD primer to be used with A01 Y-adapter), and 10 μl of previously purified DNA. The PCR program was 1 cycle of 95 °C for 5 min 30 cycles of 95 °C for 30 s, 69 °C for 1 min, and 72 °C for 30 s; 1 cycle at 72 °C for 5 min; and 4 °C hold. PCR products were purified using 0.7x AMPure beads (Beckman Coulter, Indianapolis, IN) and resuspended in 15 μl of elution buffer (Qiagen, Hilden, Germany).

NGS libraries were prepared by PCR in the following mix (amounts per sample): 5.4 μl of nuclease-free water (Life Technologies, Waltham, MA), 3 μl of 10x buffer for Taq Polymerase (MgCl_2_-free; Invitrogen, Carlsbad, CA), 0.6 μl of 10 mM dNTP mix (Thermo Fisher Scientific, Waltham, MA), 1.2 μl of 50 mM MgCl_2_ (Invitrogen, Carlsbad, CA), 0.3 μl of 5 U/μl Platinum Taq polymerase (Invitrogen, Carlsbad, CA), 1 μl of 10 μM GSP_ITR3 primer, 1.5 μl of 0.5 M TMAC (Sigma-Aldrich, St. Louis, MO), 0.5 μl of 10 μM A01-A16_P5_FWD primer with the primer number matching the adapter number, 1.5 μl of 10 μM p701–16 primers, and 15 μl of previously purified DNA (including the AMPure beads used in the previous PCR purification step). The PCR program was 1 cycle of 95 °C for 5 min; 10 cycles of 95 °C for 30 s, 75 °C for 2 min (− 1 °C/cycle), and 72 °C for 30 s; 15 cycles of 95 °C for 30 s, 69 °C for 1 min, and 72 °C for 30 s; 1 cycle at 72 °C for 5 min; and 4 °C hold. PCR products were purified using 0.7x AMPure beads (Beckman Coulter, Indianapolis, IN), and resuspended in 25 μl of elution buffer. Dual-indexed sequencing libraries were sequenced on an Illumina MiSeq cartridge (MiSeq® v2 RGT Kit 300 cyc PE-Bx 1 of 2; San Diego, CA), generating 2 × 150 bp paired-end reads.

Sample demultiplexing and unique molecular identifier (UMI) tagging was performed on raw fastq files using Je [39], allowing for up to one mismatch on either index. Read pairs in which read 2 begins with the designed primer sequence, plus an additional flanking 20 bp of the AAV2 ITR sequence (Fig. 1), were identified using FASTX barcode slitter (http://hannonlab.cshl.edu/fastx_toolkit/, allowing for up to five mismatches), fastq-pair (https://github.com/linsalrob/EdwardsLab/), and FASTP [40]. Selected read pairs were mapped to the reference genome for each sample (MM10 for mouse and RheMac8 for rhesus macaque samples) using NovoAlign (Novocraft, Selangor, Malaysia). UMI read consolidation was subsequently performed using Je [39], resulting in a UMI-consolidated BAM file. Chimeric reads spanning the ITR-genomic DNA insertion site were identified by determining split-read junctions for each read using SE-MEI (https://github.com/dpryan79/SE-MEI). Soft-clipped portions of reads were then mapped to the AAV2 reference genome using NovoAlign (Novocraft, Selangor, Malaysia). Only those originally mapped reads that were found to contain a soft-clipped read portion mapping to the AAV2 ITR with a mapping quality value greater than or equal to 30 were used to identify ITR integration sites by merging ITR integration sites found within a 50 bp window into a single ITR integration site using BEDtools [41]. Only those sites with an ITR integrated in both the forward and negative strand orientations were considered to be an identified off-target site. The genomic DNA sequence underlying each identified ITR integration site was aligned pairwise with the on-target DNA sequence motif from both the negative and positive strand orientations using the EMBOSS program [42] for semi-global alignment; this allowed us to assess sequence homology and predict the nuclease target sequence for each ITR integration site.

## Supplementary information

**Additional file1 **Supporting Dataset 1. Supporting Dataset 2. **S1 Table**. Validating ITR-Seq-identified off-target events. **S2 Table**. ITR-Seq rank of GUIDE-Seq-identified off-target events. **S3 Table.** Annotation of ITR-Seq-identified on- and off-target events. **S4 Table.** List of primer sequences used in this study.

**Additional file 2 Supplemental Figure 1**. Frequency of AAV integration in the on- and off-target sites. The number of ITR-Seq reads for the on and off-target sites are shown as a percentage of the total number of ITR-Seq reads before the filtering step (see Methods). Analysis was performed on the ITR-Seq results for liver biopsies at d17 and d128 from non-human primates treated with the indicated nuclease and AAV dose.

**Additional file 3 Supplemental Figure 2**. Distribution of mismatches between the target sequence and identified off-target sequences. Off-targets sequences were extracted from the ITR-Seq results for AAV-M1PCSK9 (at a dose of 3 × 10^13^ or 6 × 10^12^ GC/Kg, panels a and b) and AAV-M2PCSK9 (6 × 10^12^ GC/kg dose, panels c and d) groups at d17. Thirty-one top-ranked (according to the number of ITR-Seq reads) off-target sequences, with a length of 22 bp and with no more than 10 mismatches, were retained for analysis. Location of the off-target sites are shown on the left and mismatches between the off- and on-target sequences are highlighted. The data to generate the WebLogo [43] shown on top were the selected off-target sequences for each group multiplied by the reported number of ITR-Seq reads (Dataset S1).

## Data Availability

Datasets S1 and S2 are available at BioProject (accession number PRJNA609560).

## Abbreviations

AAV: Adeno-associated viral
BLESS: Breaks labeling, enrichment on streptavidin and sequencing
BLISS: Breaks labeling in situ and sequencing
CRISPR: Clusters of regularly interspersed short palindromic repeats
DNA: Deoxyribonucleic acid
dNTP: Deoxyribonucleotide triphosphate
DSB: Double-stranded breaks
EMBOSS: European Molecular Biology Open Software Suite
GC: Genome copies
hFIX: Human coagulation factor IX
ITR: Inverted terminal repeat
ITR-Seq: ITR sequencing
NGS: Next-generation sequencing
nrLAM-PCR: Non-restrictive linear-amplification mediated PCR
PBMC: Peripheral blood mononuclear cell
PCR: Polymerase chain reaction
PCSK9: Proprotein convertase subtilisin/kexin type 9
RBE: Rep-binding element
RNA: Ribonucleic acid
sgRNA: Single-guide RNA
TALENS: Transcription activator-like effector nucleases
TMAC: Tetramethylammonium chloride solution
TRS: Terminal resolution site
TSS: Transcription start sites
TTS: Transcription termination sites
UMI: Unique molecular identifier
