# Correction to: ITR-Seq, a next-generation sequencing assay, identifies genome-wide DNA editing sites in vivo following adeno-associated viral vector-mediated genome editing

**DOI:** 10.1186/s12864-020-07039-2

**Published:** 2020-11-20

**Authors:** Camilo Breton, Peter M. Clark, Lili Wang, Jenny A. Greig, James M. Wilson

**Affiliations:** Gene Therapy Program, University of Pennsylvania Perelman School of Medicine, 125 South 31st Street, Suite 1200, Philadelphia, PA 19104 USA

**Correction to: BMC Genomics 21, 239 (2020)**

**https://doi.org/10.1186/s12864-020-6655-4**

Following publication of the original article [1], we noticed that incorrect datasets were used for some analyses. We have corrected Figs. 2, 3, S1, and S2, Table S2, and dataset S1. Access to additional supporting data files via the webpage has also been restored. Importantly, the conclusions of the manuscript are not affected by this error.

**Fig. 2:**
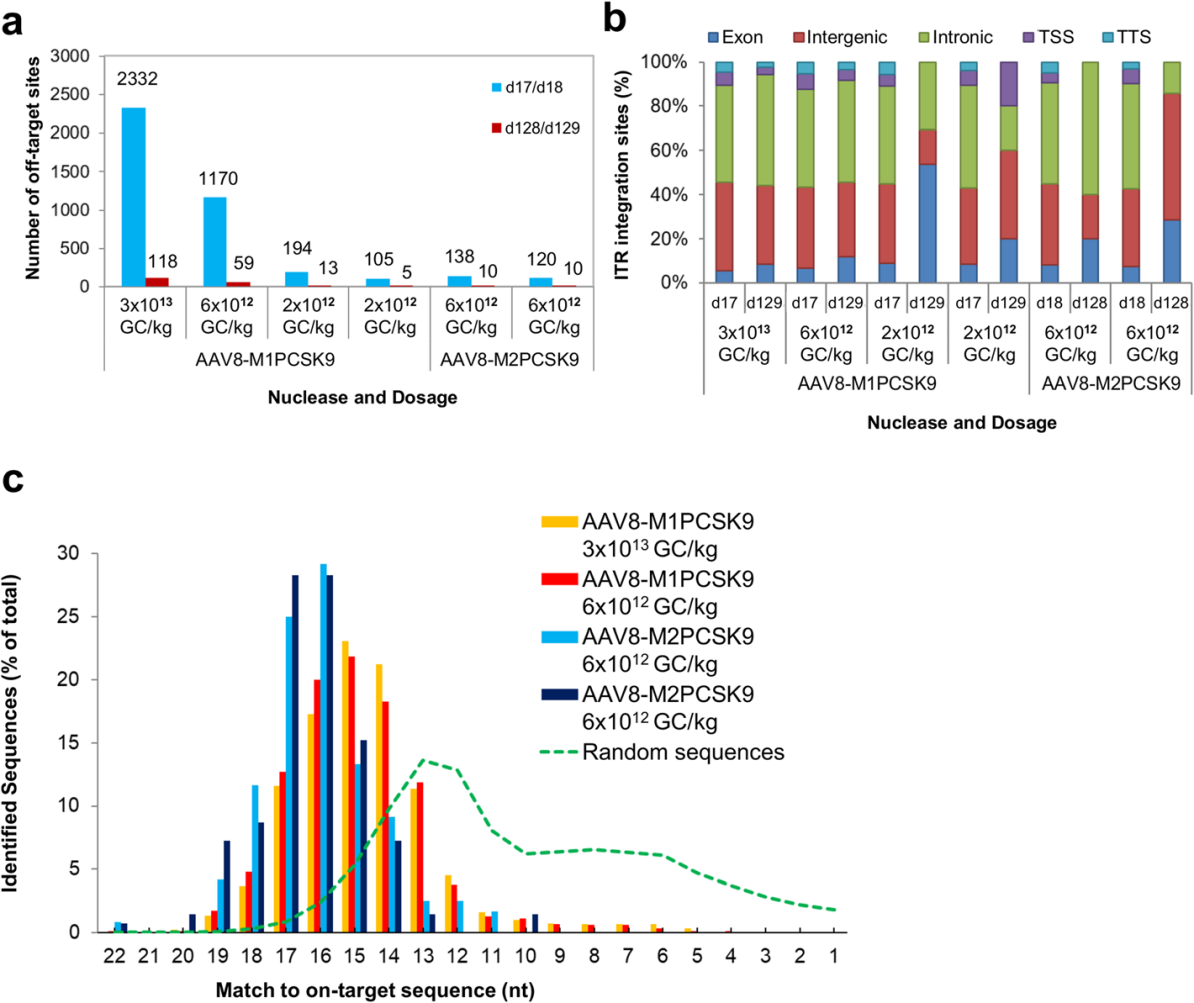
Analyzing on- and off-target activity of AAV8-M1PCSK9 and AAV8-M2PCSK9 in vivo. **a** ITR-Seq-identified integration sites in liver samples treated with AAV8-M1PCSK9 and AAV8-M2PCSK9. Samples were collected on day 17/18 and 128/129 following vector administration. **b** Functional annotation of ITR-identified integration sites. Here, we show the number of sites within exons, introns, intergenic regions, transcription start sites (TSS), and transcription termination sites (TTS). **c.** Distribution of ITR-integration sites on days 17/18 for two animals treated with either M1PCSK9 or M2PCSK9 (colored bars). Computationally generated random DNA sequences are represented by the green dotted line and are based on the number of nucleotides that match the intended target sequence (represented as a percent of all identified sites)

**Fig. 3:**
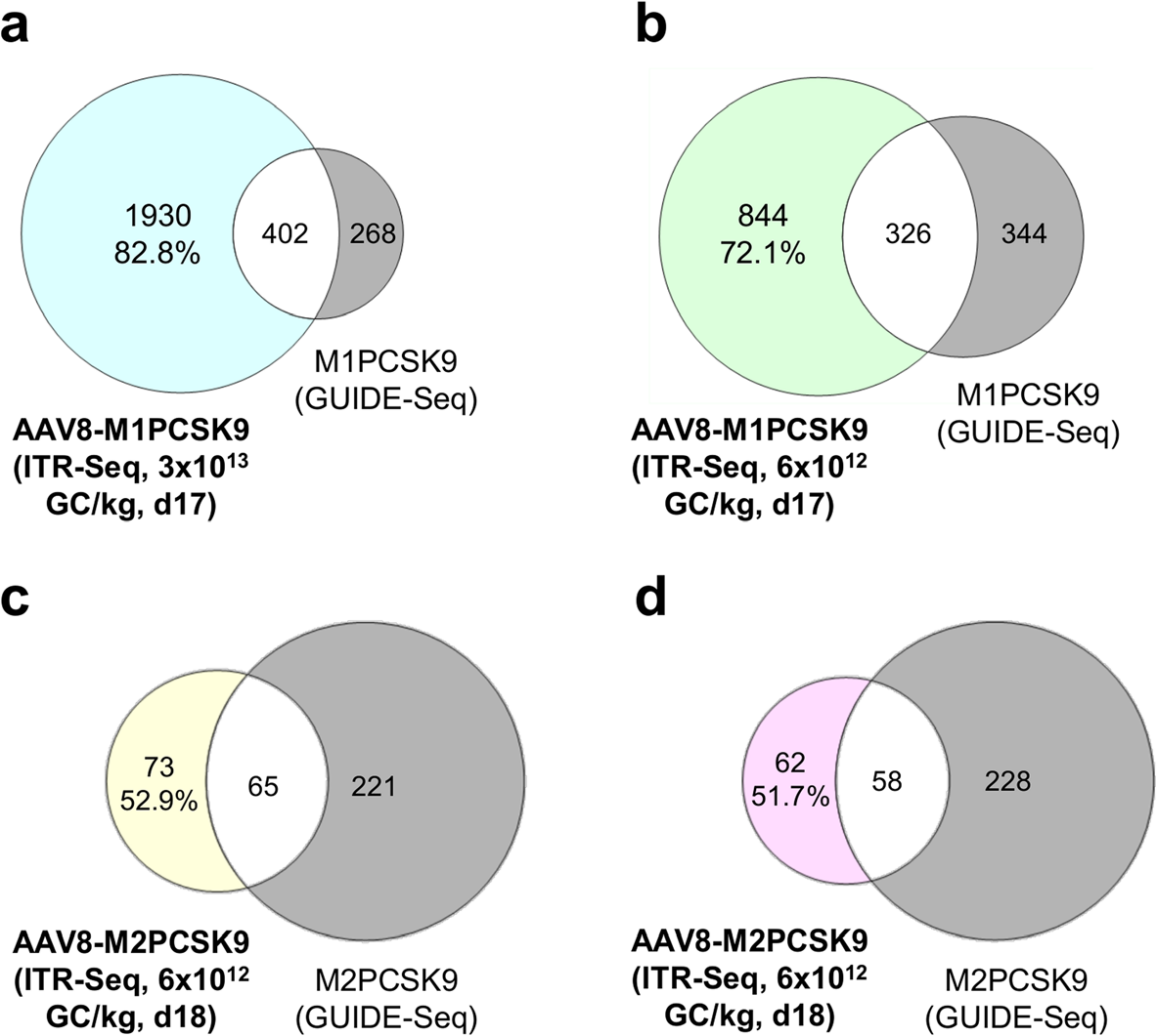
Comparing GUIDE-Seq and ITR-Seq in terms of off-target identification. Sample set intersections of identified target sites obtained from in vivo ITR-Seq (coloured) from two doses of AAV8-M1PCSK9 (3 × 10^13^ GC/kg; panel **a**; and 6 × 10^12^ GC/kg; panel **b**) or one dose of AAV8-M2PCSK9 (6 × 10^12^ GC/kg; panels **c** and **d**). We obtained target sites on day 17 post-AAV administration. In vitro GUIDE-Seq for M1PCSK9 or M2PCSK9 is shown in gray. Off-target sites identified by ITR-Seq but not by GUIDE-Seq (coloured sections) are indicated as a percent of the total number of off-target sites that were identified by in vivo ITR-Seq. White sections of the Venn diagrams show the proportion of off-target sites that were identified by both ITR-Seq and GUIDE-Seq

The corrected figures, data files and a summary of the corrections (Additional file 5) to the text have been included with this Correction article.

## Supplementary information

**Additional file 1: Figure S1.** Frequency of AAV integration in the on- and off-target sites. The number of ITR-Seq reads for the on and off-target sites are shown as a percentage of the total number of ITR-Seq reads before the filtering step (see Methods). Analysis was performed on the ITR-Seq results for liver biopsies at d17/d18 and d128/d129 from non-human primates treated with the indicated nuclease and AAV dose.

**Additional file 2: Figure S2.** Distribution of mismatches between the target sequence and identified off-target sequences. Off-targets sequences were extracted from the ITR-Seq results for AAV-M1PCSK9 (at a dose of 3 × 10^13^ or 6 × 10^12^ GC/kg, panels a and b) and AAV-M2PCSK9 (6 × 10^12^ GC/kg dose, panels c and d) groups at d17/d18. Thirty-one top-ranked (according to the number of ITR-Seq reads) off-target sequences, with a length of 22 bp and with no more than 10 mismatches, were retained for analysis. Location of the off-target sites are shown on the left and mismatches between the off- and on-target sequences are highlighted. The data to generate the WebLogo (43) shown on top were the selected off-target sequences for each group multiplied by the reported number of ITR-Seq reads (Dataset S1).

**Additional file 3: Table S2**. ITR-Seq rank of GUIDE-Seq-identified off-target events.

**Additional file 4: Dataset S1.**

**Additional file 5:** Summary of Corrections.
