# Supplementary material for: ITR-Seq, a next-generation sequencing assay, identifies genome-wide DNA editing sites in vivo following adeno-associated viral vector-mediated genome editing
Source: BMC Genomics. 2020 Mar 17;21:239. doi: 10.1186/s12864-020-6655-4 (PMC7076944; doi:10.1186/s12864-020-6655-4)
Supplement: Supplementary file 1 — Additional file1 Supporting Dataset 1. Supporting Dataset 2. S1 Table. Validating ITR-Seq-identified off-target events. S2 Table. ITR-Seq rank of GUIDE-Seq-identified off-target events. S3 Table. Annotation of ITR-Seq-identified on- and off-target events. S4 Table. List of primer sequences used in this study. [file 12864_2020_6655_MOESM1_ESM.docx]

**SUPPLEMENTAL INFORMATION**

**Table of Contents**

1. Supporting Dataset 1
2. Supporting Dataset 2
3. S1 Table
4. S2 Table
5. S3 Table
6. S4 Table

**S1 Dataset**

ITR-Seq-identified on- and off-target events in DNA samples from rhesus macaques harvested at d17/18 and d128/129 post-treatment with AAV8-M1PCSK9 and AAV8-MPCSK9 at different doses. The dataset is available at BioProject (accession number PRJNA609560).

**S2 Dataset**

GUIDE-Seq identified off-target sites for M1PCSK9 and M2PCSK9 in LLC-MK2 cells. N = 2 experiments. The dataset is available at BioProject (accession number PRJNA609560).

**S1 Table. Validating ITR-Seq-identified off-target events.** We randomly selected ITR-Seq-identified off-target events from the ITR-Seq results for d18 liver samples from macaques treated with AAV8-M1PCSK9 at a dose of 3x10^13^ or 6x10^12^ GC/kg. We investigated ITR integration (ITR-containing reads) and indel percentage in these selected loci by AMP-Seq. Bold typeface indicates the locus where no ITR-containing reads were identified. N.I. Off-target not identified by ITR-Seq in that particular macaque.

| **RA1866 (AAV8.M1PCSK9 3x10¹³ GC/kg) d18** | | | | | | | | |
| --- | --- | --- | --- | --- | --- | --- | --- | --- |
| **Location** | **ITR-Seq  rank** | **Positive strand** | | |  | **Negative strand** | | |
|  |  | **Mapped  reads** | **ITR-containing  reads (%)** | **Indel%** |  | **Mapped  reads** | **ITR-containing  reads (%)** | **Indel%** |
| 1:54708760-54709149 | 1 | 9983 | 268 (2.68%) | 4.19% |  | 1822 | 169 (9.28%) | 14.05% |
| 2:47193033-47193289 | 26 | 3052 | 82 (2.69%) | 3.93% |  | 1473 | 19 (1.29%) | 2.38% |
| 10:92093017-92093261 | 45 | 1538 | 48 (3.12%) | 3.97% |  | 428 | 3 (0.7%) | 1.17% |
| 13:92560640-92560913 | 68 | 6445 | 296 (4.59%) | 6.16% |  | 1234 | 13 (1.05%) | 3.16% |
| 7:167714074-167714330 | 71 | 4110 | 7 (0.17%) | 0.75% |  | 1112 | 2 (0.18%) | 0.90% |
| 14:11716288-11716580 | 99 | 749 | 2 (0.27%) | 0.53% |  | 1872 | 11 (0.59%) | 0.85% |
| 12:101681595-101681841 | 106 | 4642 | 93 (2%) | 2.33% |  | 877 | 6 (0.68%) | 0.80% |
| 12:122915957-122916217 | 138 | 5474 | 32 (0.58%) | 0.91% |  | 1886 | 10 (0.53%) | 1.01% |
| 11:48393553-48393784 | 289 | 5933 | 11 (0.19%) | 1.03% |  | 1215 | 4 (0.33%) | 2.22% |
| 7:49930168-49930416 | 307 | 3777 | 6 (0.16%) | 0.32% |  | 723 | 1 (0.14%) | 1.24% |
| 12:123664517-123664748 | 320 | 2223 | 19 (0.85%) | 1.08% |  | 10 | 0 (0%) | 0.00% |
| 11:55961852-55962103 | 335 | 4118 | 4 (0.1%) | 0.32% |  | 814 | 3 (0.37%) | 0.37% |
| 3:61235495-61235760 | 370 | 8205 | 25 (0.3%) | 0.69% |  | 2248 | 7 (0.31%) | 0.80% |
| 10:885907-886142 | 376 | 6205 | 42 (0.68%) | 0.71% |  | 1931 | 6 (0.31%) | 0.31% |
| 5:2440078-2440309 | 390 | 3587 | 11 (0.31%) | 0.86% |  | 707 | 5 (0.71%) | 1.70% |
| 7:167909341-167909569 | 455 | 5266 | 5 (0.09%) | 0.19% |  | 134 | 1 (0.75%) | 0.75% |
| **10:91314288-91314527** | 547 | 168 | **0 (0%)** | **0.00%** |  | 1001 | **0 (0%)** | **0.30%** |
| 12:56755578-56755807 | 590 | 4785 | 36 (0.75%) | 0.94% |  | 1870 | 0 (0%) | 0.27% |
| **7:165842063-165842313** | 1094 | 29939 | **0 (0%)** | **18.22%** |  | 371 | **0 (0%)** | **3.50%** |
| 12:125235524-125235749 | 1119 | 2030 | 1 (0.05%) | 0.15% |  | 557 | 1 (0.18%) | 0.36% |
| 4:44583987-44584212 | 1153 | 4056 | 2 (0.05%) | 0.07% |  | 1692 | 1 (0.06%) | 0.30% |
| 10:27183178-27183404 | 1253 | 3461 | 0 (0%) | 0.20% |  | 2291 | 1 (0.04%) | 0.35% |
| 10:79726682-79726923 | 1260 | 6966 | 3 (0.04%) | 0.27% |  | 1651 | 1 (0.06%) | 0.24% |
| **16:19823284-19823518** | 1288 | 3583 | **0 (0%)** | **0.14%** |  | 825 | **0 (0%)** | **0.00%** |
| 18:39311584-39311811 | 1696 | 6523 | 24 (0.37%) | 0.78% |  | 1829 | 0 (0%) | 0.16% |
| 20:359062-359285 | N.I. | 5270 | 39 (0.74%) | 0.85% |  | 1701 | 1 (0.06%) | 0.24% |
| 7:165269225-165269449 | N.I. | 5985 | 11 (0.18%) | 0.42% |  | 1752 | 0 (0%) | 0.23% |
|  |  |  |  |  |  |  |  |  |
| **RA1857 (AAV8.M1PCSK9 6x10¹² GC/kg) d18** | | | | | | | | |
| **Location** | **ITR-Seq  rank** | **Positive strand** | | |  | **Negative strand** | | |
|  |  | **Mapped  reads** | **ITR-containing  reads (%)** | **Indel%** |  | **Mapped  reads** | **ITR-containing  reads (%)** | **Indel%** |
| 1:54708760-54709149 | 1 | 12320 | 223 (1.81%) | 4.52% |  | 2020 | 114 (5.64%) | 10.59% |
| 2:47193033-47193289 | 24 | 3420 | 36 (1.05%) | 1.40% |  | 2632 | 13 (0.49%) | 1.10% |
| 13:92560640-92560913 | 68 | 4619 | 11 (0.24%) | 1.82% |  | 1032 | 3 (0.29%) | 2.03% |
| 10:92093017-92093261 | 73 | 2742 | 8 (0.29%) | 0.69% |  | 538 | 7 (1.3%) | 1.30% |
| 12:101681595-101681841 | 98 | 9377 | 21 (0.22%) | 0.34% |  | 753 | 4 (0.53%) | 0.53% |
| 14:11716288-11716580 | 112 | 1049 | 1 (0.1%) | 0.29% |  | 1548 | 4 (0.26%) | 0.58% |
| 12:122915957-122916217 | 113 | 7067 | 271 (3.83%) | 4.02% |  | 1795 | 3 (0.17%) | 0.22% |
| 7:167909341-167909569 | 165 | 7711 | 5 (0.06%) | 0.10% |  | 1260 | 0 (0%) | 0.00% |
| 11:55961852-55962103 | 170 | 6107 | 3 (0.05%) | 0.10% |  | 1336 | 1 (0.07%) | 0.22% |
| 7:167714074-167714330 | 193 | 5611 | 0 (0%) | 0.43% |  | 1090 | 1 (0.09%) | 0.64% |
| 12:123664517-123664748 | 240 | 3104 | 1 (0.03%) | 0.10% |  | 14 | 0 (0%) | 0.00% |
| 11:48393553-48393784 | 249 | 8043 | 1 (0.01%) | 0.50% |  | 1009 | 4 (0.4%) | 0.79% |
| **7:49930168-49930416** | 264 | 3956 | **0 (0%)** | **0.13%** |  | 543 | **0 (0%)** | **0.00%** |
| 10:885907-886142 | 279 | 7861 | 5 (0.06%) | 0.23% |  | 1838 | 1 (0.05%) | 0.44% |
| 3:61235495-61235760 | 403 | 9571 | 2 (0.02%) | 0.14% |  | 1668 | 1 (0.06%) | 0.36% |
| **7:165269225-165269449** | 546 | 8373 | **0 (0%)** | **0.21%** |  | 1559 | **0 (0%)** | **0.19%** |
| 18:39311584-39311811 | 652 | 9207 | 0 (0%) | 0.17% |  | 2859 | 1 (0.03%) | 0.31% |
| **20:359062-359285** | 688 | 7205 | **0 (0%)** | **0.14%** |  | 1625 | **0 (0%)** | **0.31%** |
| 10:27183178-27183404 | 777 | 5667 | 0 (0%) | 0.26% |  | 2004 | 1 (0.05%) | 0.45% |
| **10:91314288-91314527** | 841 | 4101 | **0 (0%)** | **0.29%** |  | 19 | **0 (0%)** | **0.00%** |
| 12:56755578-56755807 | 990 | 6349 | 5 (0.08%) | 0.20% |  | 1720 | 2 (0.12%) | 0.41% |
| 10:79726682-79726923 | 1034 | 9693 | 2 (0.02%) | 0.24% |  | 1564 | 0 (0%) | 0.19% |
| 16:19823284-19823518 | 1055 | 5279 | 0 (0%) | 0.09% |  | 1107 | 2 (0.18%) | 0.27% |
| 5:2440078-2440309 | N.I. | 4475 | 4 (0.09%) | 0.45% |  | 1214 | 0 (0%) | 0.25% |
| **7:165842063-165842313** | N.I. | 41062 | **0 (0%)** | **16.04%** |  | 360 | **0 (0%)** | **1.39%** |
| **12:125235524-125235749** | N.I. | 2652 | **0 (0%)** | **0.04%** |  | 487 | **0 (0%)** | **0.00%** |
| 4:44583987-44584212 | N.I. | 4812 | 4 (0.08%) | 0.12% |  | 1373 | 0 (0%) | 0.00% |

**S2 Table. ITR-Seq rank of GUIDE-Seq-identified off-target events.**

GUIDE-Seq-identified off-target events, validated by amplicon sequencing analysis on days 17/18 in liver samples obtained from macaques treated with AAV8-M1PCSK9 or AAV8-M2PCSK9 at the indicated doses^24^ and their corresponding ITR-Seq rank. Bold typeface indicates the off-target events in which the reported indel percentage quantified by amplicon sequencing was significantly higher than untreated controls. N.I., not identified by ITR-Seq.

| **Nuclease:** | **AAV8-M1PCSK9** | | | |  | **AAV8-M2PCSK9** | |
| --- | --- | --- | --- | --- | --- | --- | --- |
| **AAV dose:** | 3x10^13^ GC/kg | 6x10^12^ GC/kg | 2x10^12^ GC/kg | 2x10^12^ GC/kg |  | 6x10^12^ GC/kg | 6x10^12^ GC/kg |
| **Id number:** | RA1866 | RA1857 | RA1829 | RA2334 |  | RA2125 | RA2343 |
| High rank |  |  |  |  | High rank |  |  |
| Chr5:112049529 | **199** | **66** | **69** | **N.I.** | Chr10:72232623 | **84** | **N.I.** |
| Chr20:69811042 | **8** | **2** | **N.I.** | **N.I.** | Chr5:112049529 | **30** | **62** |
| Chr7:123575698 | **N.I.** | N.I. | N.I. | N.I. | Chr19:51609207 | N.I. | N.I. |
| Chr12:10658914 | **157** | **92** | **N.I.** | **N.I.** | Chr19:31971930 | **63** | **N.I.** |
| Chr12:51647755 | **90** | **37** | **N.I.** | **N.I.** | Chr16:48383076 | **14** | **6** |
| Chr13:92389310 | **41** | **58** | **N.I.** | **N.I.** | Chr16:41164165 | N.I. | N.I. |
| Chr16:49265525 | **2** | **5** | **5** | **2** | Chr9:53019653 | **11** | **19** |
| Chr13:43000760 | **6** | **4** | **57** | **50** | Chr14:11716320 | **80** | N.I. |
| Chr6:2022570 | **34** | **21** | **N.I.** | **37** | Chr14:69311382 | **13** | **7** |
| Chr5:139700784 | **12** | **N.I.** | N.I. | **N.I.** | Chr7:123575698 | N.I. | N.I. |
| Chr9:114398062 | **14** | **11** | **N.I.** | **N.I.** | Chr3:169340141 | N.I. | N.I. |
| Chr9:53019653 | **9** | **17** | **4** | **4** | Chr5:178494103 | N.I. | N.I. |
| Chr10:22429622 | **23** | **16** | **N.I.** | **96** | Chr12:51647755 | **36** | **N.I.** |
| Chr12:46743016 | **19** | **38** | **N.I.** | **N.I.** | Chr16:49265525 | **2** | **4** |
| Chr10:72232623 | N.I. | **28** | **6** | **9** | Chr6:2022570 | **22** | **9** |
| Low rank |  |  |  |  | Low rank |  |  |
| Chr7:167169127 | **1242** | N.I. | N.I. | N.I. | Chr19:5020267 | N.I. | N.I. |
| Chr11:102820504 | **1476** | **N.I.** | **N.I.** | 135 | Chr3:75646089 | **N.I.** | N.I. |
| Chr1:62000776 | **1289** | N.I. | N.I. | N.I. | Chr14:13573718 | **N.I.** | N.I. |
| Chr18:44590418 | N.I. | N.I. | N.I. | N.I. | Chr10:91307567 | **N.I.** | N.I. |
| Chr15:80157785 | N.I. | **N.I.** | N.I. | **N.I.** | Chr1:139404583 | N.I. | N.I. |
| Chr7:119527355 | N.I. | N.I. | N.I. | N.I. | Chr12:30957735 | N.I. | N.I. |

**S3 Table. Annotation of ITR-Seq-identified on- and off-target events.**

Rank-ordered list of identified nuclease target sites.

| **Sample (dose GC)** |  | **Chromosome** | **Start** | **Stop** | **ITR-Seq  reads** | **Gene Symbol** | **Match** |
| --- | --- | --- | --- | --- | --- | --- | --- |
| AAV8-SaCas9 (3x10¹¹) + AAV8-sgRNA1 (2x10¹²) | Mouse A | 2 | 31518669 | 31518688 | 2967 | Ass1 | **11/20** |
|  |  | 2 | 98667069 | 98667090 | 64 | Gm10800 | 12/20 |
|  |  | 3 | 98132713 | 98132733 | 31 | Notch2 | 19/20 |
|  |  | 13 | 62868971 | 62868992 | 10 | Fbp1 | 13/20 |
| AAV8-SaCas9 (3x10¹¹) + AAV8-sgRNA1 (2x10¹²) | Mouse B | 2 | 31518638 | 31518658 | 6439 | Ass1 | **20/20** |
|  |  | 3 | 98132713 | 98132725 | 36 | Notch2 | 12/20 |
|  |  | 5 | 90467666 | 90467685 | 5 | Alb | 12/20 |
| AAV8-LbCpf1 (3x10¹¹) + AAV8-sgRNA1 (2x10¹²) | Mouse A | 2 | 31518479 | 31518502 | 627 | Ass1 | **23/23** |
| AAV8-LbCpf1 (3x10¹¹) + AAV8-sgRNA1 (2x10¹²) | Mouse B | 2 | 31518479 | 31518502 | 618 | Ass1 | **23/23** |
|  |  | 2 | 98667141 | 98667169 | 25 | Gm10800 | 19/23 |
|  |  | 3 | 129856572 | 129856596 | 4 | Cfi | 21/23 |
| AAV8-LbCpf1 (3x10¹¹) + AAV8-sgRNA2 (2x10¹²) | Mouse A | 2 | 31519011 | 31519034 | 1006 | Ass1 | **23/23** |
|  |  | 5 | 90474812 | 90474837 | 4 | Alb | 14/23 |
|  |  | 2 | 98666971 | 98666997 | 3 | Gm10800 | 17/23 |
| AAV8-LbCpf1 (3x10¹¹) + AAV8-sgRNA2 (2x10¹²) | Mouse B | 2 | 31519011 | 31519034 | 572 | Ass1 | **23/23** |
|  |  | 2 | 98667132 | 98667155 | 18 | Gm10800 | 15/23 |
|  |  | 5 | 90509831 | 90509858 | 6 | Alb | 17/23 |
|  |  | 14 | 20208435 | 20208460 | 2 | Gm43449 | 15/23 |
| AAV8-AsCpf1 (3x10¹¹) + AAV8-sgRNA1 (2x10¹²) | Mouse A | 2 | 31518479 | 31518502 | 151 | Ass1 | **23/23** |
|  |  | 2 | 98667150 | 98667169 | 18 | Gm10800 | 13/23 |
|  |  | 5 | 90474820 | 90474842 | 5 | Alb | 12/23 |
|  |  | 5 | 90499474 | 90499500 | 5 | Afp | 14/23 |
| AAV8-AsCpf1 (3x10¹¹) + AAV8-sgRNA1 (2x10¹²) | Mouse B | 2 | 31518479 | 31518502 | 104 | Ass1 | **23/23** |
|  |  | 5 | 90474848 | 90474864 | 5 | Alb | 9/23 |
| AAV8-AsCpf1 (3x10¹¹) + AAV8-sgRNA2 (2x10¹²) | Mouse A | 2 | 98667153 | 98667174 | 7 | Gm10800 | 14/23 |
|  |  | 17 | 39848307 | 39848315 | 5 | CT010467.1 | 5/23 |
| AAV8-AsCpf1 (3x10¹¹) + AAV8-sgRNA2 (2x10¹²) | Mouse B | 2 | 31519052 | 31519078 | 36 | Ass1 | 17/23 |
|  |  | 11 | 7201439 | 7201456 | 2 | Igfbp1 | 10/23 |
| AAV8-SaCas9 (10¹¹) + AAV8-sgRNA1 (10¹²) | Mouse A | 2 | 31518638 | 31518658 | 8990 | ASS1 | **20 / 20** |
|  |  | 16 | 22897639 | 22897660 | 13 | Ahsg | 11 / 20 |
|  |  | 11 | 7202377 | 7202398 | 9 | Igfbp1 | 12 / 20 |
|  |  | 5 | 90476373 | 90476394 | 9 | Alb | 14 / 20 |
| AAV8-SaCas9 (10¹¹) + AAV8-sgRNA1 (10¹²) | Mouse B | 2 | 31518638 | 31518658 | 14675 | ASS1 | **20 / 20** |
|  |  | 2 | 98667069 | 98667090 | 77 | Gm10800 | 12 / 20 |
|  |  | 11 | 7201449 | 7201458 | 35 | Igfbp1 | 7 / 20 |
|  |  | 16 | 22895229 | 22895247 | 20 | Ahsg | 12 / 20 |
|  |  | 5 | 90465786 | 90465801 | 16 | Alb | 11 / 20 |
|  |  | 11 | 101372382 | 101372394 | 13 | G6pc | 8 / 20 |
| AAV8-SaCas9 (3x10¹¹) + AAV8-sgRNA1 (10¹²) | Mouse A | 2 | 31518638 | 31518658 | 20860 | ASS1 | **20 / 20** |
|  |  | 2 | 98667242 | 98667259 | 76 | Gm10800 | 12 / 20 |
|  |  | 11 | 7201449 | 7201464 | 27 | Igfbp1 | 12 / 20 |
|  |  | 5 | 90507403 | 90507420 | 9 | Alb | 12 / 20 |
| AAV8-SaCas9 (3x10¹¹) + AAV8-sgRNA1 (10¹²) | Mouse B | 2 | 31518638 | 31518658 | 9877 | ASS1 | **20 / 20** |
|  |  | 2 | 98666969 | 98666987 | 8 | Gm10800 | 12 / 20 |
|  |  | 16 | 22897639 | 22897660 | 7 | Ahsg | 11 / 20 |
| AAV8-LbCpf1 (10¹¹) + AAV8-sgRNA2 (10¹²) | Mouse A | 2 | 31519011 | 31519034 | 584 | ASS1 | **23 / 23** |
|  |  | 2 | 98667191 | 98667217 | 113 | Gm10800 | 16 / 23 |
|  |  | 6 | 103649175 | 103649198 | 35 | Chl1 | 12 / 23 |
|  |  | 5 | 90474812 | 90474837 | 20 | Alb | 14 / 23 |
| AAV8-LbCpf1 (10¹¹) + AAV8-sgRNA2 (10¹²) | Mouse B | 2 | 31519011 | 31519034 | 467 | ASS1 | **23 / 23** |
|  |  | 2 | 98667132 | 98667155 | 69 | Gm10800 | 15 / 23 |
|  |  | 5 | 90474789 | 90474799 | 20 | Alb | 8 / 23 |
|  |  | 11 | 7201415 | 7201439 | 15 | Igfbp1 | 14 / 23 |
|  |  | 16 | 84964041 | 84964061 | 5 | App | 13 / 23 |
| AAV8-LbCpf1 (3x10¹¹) + AAV8-sgRNA2 (10¹²) | Mouse A | 2 | 31519011 | 31519034 | 2293 | ASS1 | **23 / 23** |
|  |  | 2 | 98667191 | 98667217 | 84 | Gm10800 | 16 / 23 |
|  |  | 5 | 90467686 | 90467710 | 36 | Alb | 14 / 23 |
|  |  | 11 | 7201434 | 7201443 | 24 | Igfbp1 | 6 / 23 |
|  |  | 7 | 73496004 | 73496027 | 13 | chd2 | 13 / 23 |
|  |  | 11 | 57076605 | 57076623 | 7 | Gria1 | 10 / 23 |
| AAV8-LbCpf1 (3x10¹¹) + AAV8-sgRNA2 (10¹²) | Mouse B | 2 | 31519011 | 31519034 | 1081 | ASS1 | **23 / 23** |
|  |  | 2 | 98667076 | 98667100 | 17 | Gm10800 | 15 / 23 |
|  |  | 5 | 90474774 | 90474799 | 15 | Alb | 17 / 23 |

**S4 Table. List of primer sequences used in this study.**

| **Name** | **Sequence (5' to 3')** |
| --- | --- |
| GSP_ITR3.AAV2 | TGACTGGAGTCCTCTCTATGGGCAGTCGGTGATACAAGGAACCCCTAGTGATGGAGTTGGCC |
| A01-P5-Fwd | AATGATACGGCGACCACCGAGATCTACACTAGATCGC |
| A02-P5-Fwd | AATGATACGGCGACCACCGAGATCTACACCTCTCTAT |
| A03-P5-Fwd | AATGATACGGCGACCACCGAGATCTACACTATCCTCT |
| A04-P5-Fwd | AATGATACGGCGACCACCGAGATCTACACAGAGTAGA |
| A05-P5-Fwd | AATGATACGGCGACCACCGAGATCTACACGTAAGGAG |
| A06-P5-Fwd | AATGATACGGCGACCACCGAGATCTACACACTGCATA |
| A07-P5-Fwd | AATGATACGGCGACCACCGAGATCTACACAAGGAGTA |
| A08-P5-Fwd | AATGATACGGCGACCACCGAGATCTACACCTAAGCCT |
| A09-P5-Fwd | AATGATACGGCGACCACCGAGATCTACACGACATTGT |
| A10-P5-Fwd | AATGATACGGCGACCACCGAGATCTACACACTGATGG |
| A11-P5-Fwd | AATGATACGGCGACCACCGAGATCTACACGTACCTAG |
| A12-P5-Fwd | AATGATACGGCGACCACCGAGATCTACACCAGAGCTA |
| A13-P5-Fwd | AATGATACGGCGACCACCGAGATCTACACCATAGTGA |
| A14-P5-Fwd | AATGATACGGCGACCACCGAGATCTACACTACCTAGT |
| A15-P5-Fwd | AATGATACGGCGACCACCGAGATCTACACCGCGATAT |
| A16-P5-Fwd | AATGATACGGCGACCACCGAGATCTACACTGGATTGT |
| p701 | CAAGCAGAAGACGGCATACGAGATTCGCCTTAGTGACTGGAGTCCTCTCTATGGGCAGTCGGTGA |
| p702 | CAAGCAGAAGACGGCATACGAGATCTAGTACGGTGACTGGAGTCCTCTCTATGGGCAGTCGGTGA |
| p703 | CAAGCAGAAGACGGCATACGAGATTTCTGCCTGTGACTGGAGTCCTCTCTATGGGCAGTCGGTGA |
| p704 | CAAGCAGAAGACGGCATACGAGATGCTCAGGAGTGACTGGAGTCCTCTCTATGGGCAGTCGGTGA |
| p705 | CAAGCAGAAGACGGCATACGAGATAGGAGTCCGTGACTGGAGTCCTCTCTATGGGCAGTCGGTGA |
| p706 | CAAGCAGAAGACGGCATACGAGATCATGCCTAGTGACTGGAGTCCTCTCTATGGGCAGTCGGTGA |
| p707 | CAAGCAGAAGACGGCATACGAGATGTAGAGAGGTGACTGGAGTCCTCTCTATGGGCAGTCGGTGA |
| p708 | CAAGCAGAAGACGGCATACGAGATCCTCTCTGGTGACTGGAGTCCTCTCTATGGGCAGTCGGTGA |
| p709 | CAAGCAGAAGACGGCATACGAGATTTACCGACGTGACTGGAGTCCTCTCTATGGGCAGTCGGTGA |
| p710 | CAAGCAGAAGACGGCATACGAGATAGTGACCTGTGACTGGAGTCCTCTCTATGGGCAGTCGGTGA |
| p711 | CAAGCAGAAGACGGCATACGAGATTCGGATTCGTGACTGGAGTCCTCTCTATGGGCAGTCGGTGA |
| p712 | CAAGCAGAAGACGGCATACGAGATCAAGGTACGTGACTGGAGTCCTCTCTATGGGCAGTCGGTGA |
| p713 | CAAGCAGAAGACGGCATACGAGATTCCTCATGGTGACTGGAGTCCTCTCTATGGGCAGTCGGTGA |
| p714 | CAAGCAGAAGACGGCATACGAGATGTCAGTCAGTGACTGGAGTCCTCTCTATGGGCAGTCGGTGA |
| p715 | CAAGCAGAAGACGGCATACGAGATCGAATACGGTGACTGGAGTCCTCTCTATGGGCAGTCGGTGA |
| p716 | CAAGCAGAAGACGGCATACGAGATTCTAGGAGGTGACTGGAGTCCTCTCTATGGGCAGTCGGTGA |
| A01 | AATGATACGGCGACCACCGAGATCTACACTAGATCGCNNWNNWNNACACTCTTTCCCTACACGACGCTCTTCCGATCT |
| A02 | AATGATACGGCGACCACCGAGATCTACACCTCTCTATNNWNNWNNACACTCTTTCCCTACACGACGCTCTTCCGATOT |
| A03 | AATGATACGGCGACCACCGAGATCTACACTATCCTCTNNWNNWNNACACTCTTTCCCTACACGACGCTCTTCCGATOT |
| A04 | AATGATACGGCGACCACCGAGATCTACACAGAGTAGANNWNNWNNACACTCTTTCCCTACACGACGCTCTTCCGATOT |
| A05 | AATGATACGGCGACCACCGAGATCTACACGTAAGGAGNNWNNWNNACACTCTTTCCCTACACGACGCTCTTCCGATOT |
| A06 | AATGATACGGCGACCACCGAGATCTACACACTGCATANNWNNWNNACACTCTTTCCCTACACGACGCTCTTCCGATOT |
| A07 | AATGATACGGCGACCACCGAGATCTACACAAGGAGTANNWNNWNNACACTCTTTCCCTACACGACGCTCTTCCGATOT |
| A08 | AATGATACGGCGACCACCGAGATCTACACCTAAGCCTNNWNNWNNACACTCTTTCCCTACACGACGCTCTTCCGATOT |
| A09 | AATGATACGGCGACCACCGAGATCTACACGACATTGTNNWNNWNNACACTCTTTCCCTACACGACGCTCTTCCGATOT |
| A10 | AATGATACGGCGACCACCGAGATCTACACACTGATGGNNWNNWNNACACTCTTTCCCTACACGACGCTCTTCCGATOT |
| A11 | AATGATACGGCGACCACCGAGATCTACACGTACCTAGNNWNNWNNACACTCTTTCCCTACACGACGCTCTTCCGATOT |
| A12 | AATGATACGGCGACCACCGAGATCTACACCAGAGCTANNWNNWNNACACTCTTTCCCTACACGACGCTCTTCCGATOT |
| A13 | AATGATACGGCGACCACCGAGATCTACACCATAGTGANNWNNWNNACACTCTTTCCCTACACGACGCTCTTCCGATOT |
| A14 | AATGATACGGCGACCACCGAGATCTACACTACCTAGTNNWNNWNNACACTCTTTCCCTACACGACGCTCTTCCGATOT |
| A15 | AATGATACGGCGACCACCGAGATCTACACCGCGATATNNWNNWNNACACTCTTTCCCTACACGACGCTCTTCCGATOT |
| A16 | AATGATACGGCGACCACCGAGATCTACACTGGATTGTNNWNNWNNACACTCTTTCCCTACACGACGCTCTTCCGATOT |
| MiSeq_common | [Phos]GATCGGAAGAGooA |
| Index1 | ATCACCGACTGCCCATAGAGAGGACTCCAGTCAC |
| Read2 | GTGACTGGAGTCCTCTCTATGGGCAGTCGGTGAT |
| P5_1 | AATGATACGGCGACCACCGAGATCTA |
| P5_2 | AATGATACGGCGACCACCGAGATCTACAC |
