# Supplementary figures and images for: ITR-Seq, a next-generation sequencing assay, identifies genome-wide DNA editing sites in vivo following adeno-associated viral vector-mediated genome editing
Source: BMC Genomics. 2020 Mar 17;21:239. doi: 10.1186/s12864-020-6655-4 (PMC7076944; doi:10.1186/s12864-020-6655-4)

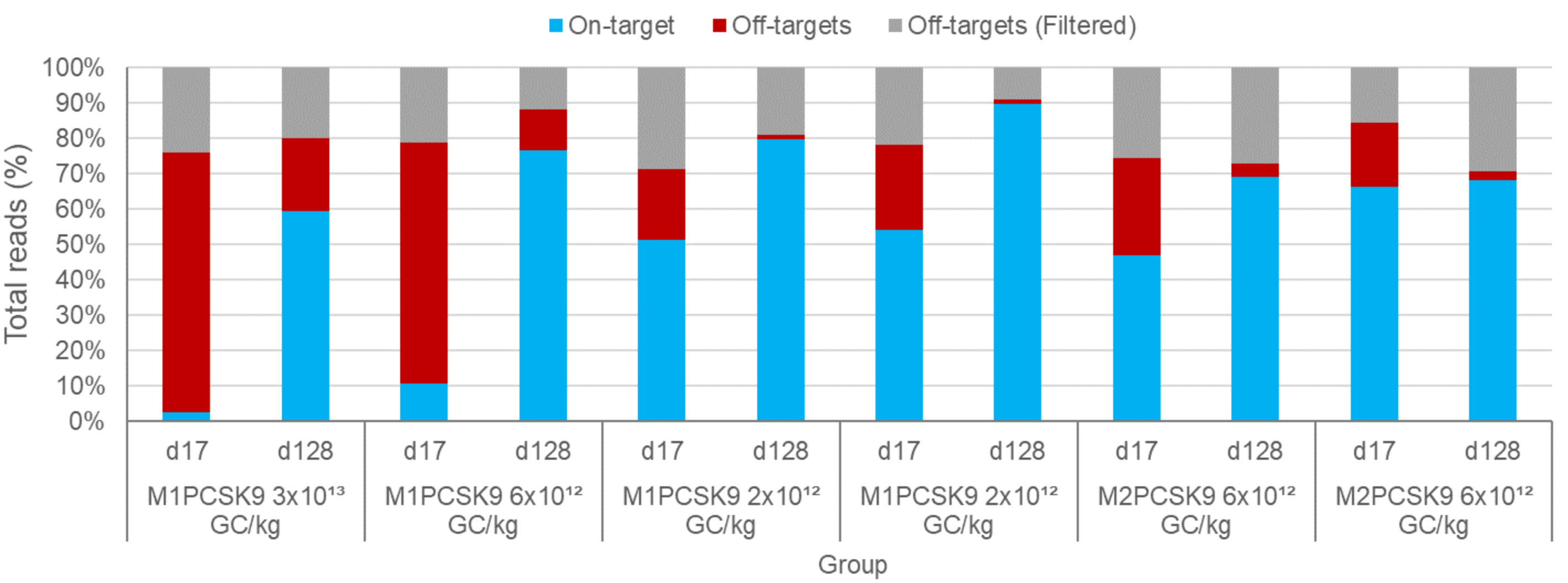

Supplement: Supplementary file 2 — Additional file 2 Supplemental Figure 1. Frequency of AAV integration in the on- and off-target sites. The number of ITR-Seq reads for the on and off-target sites are shown as a percentage of the total number of ITR-Seq reads before the filtering step (see Methods). Analysis was performed on the ITR-Seq results for liver biopsies at d17 and d128 from non-human primates treated with the indicated nuclease and AAV dose. [file 12864_2020_6655_MOESM2_ESM.tif]

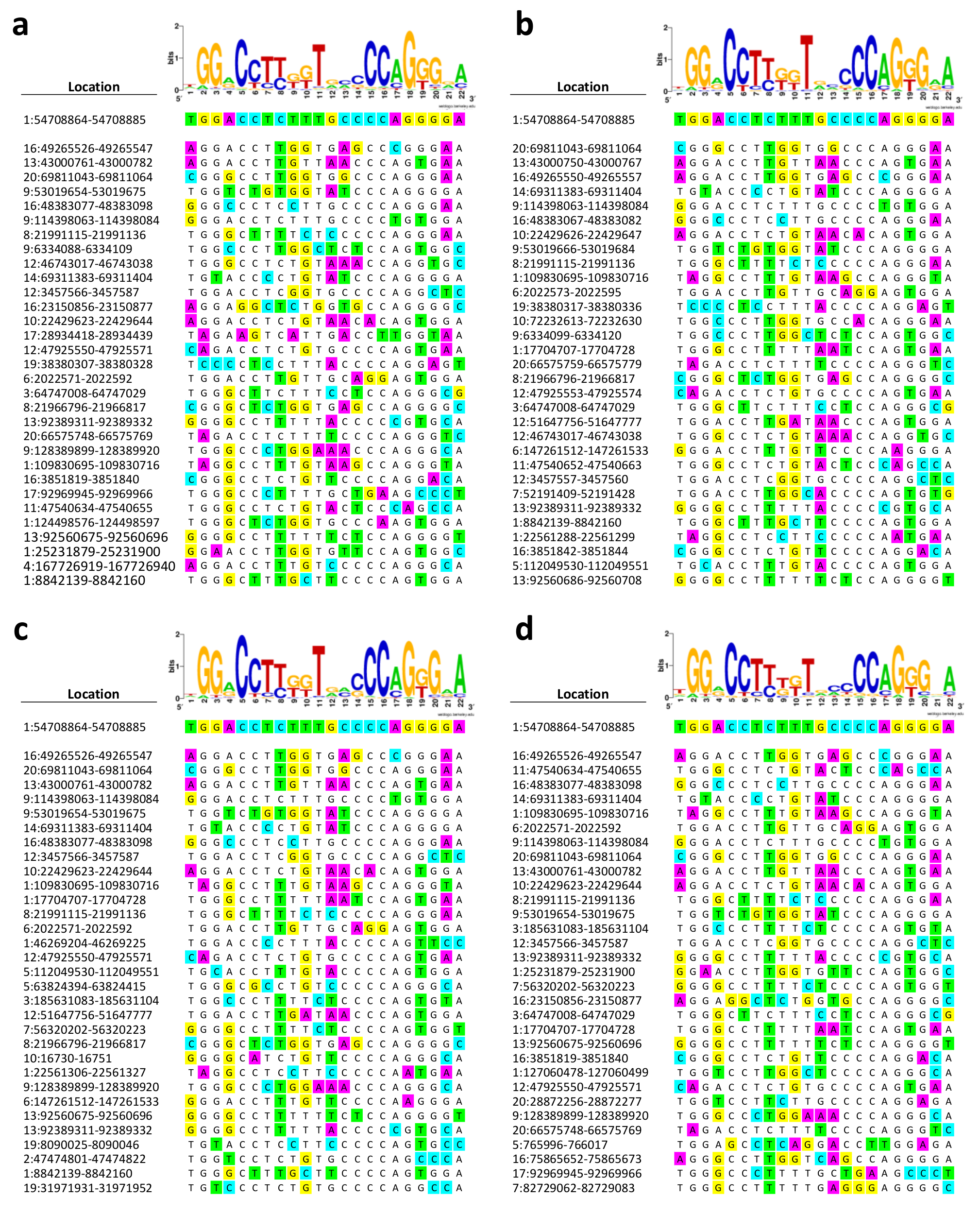

Supplement: Supplementary file 3 — Additional file 3 Supplemental Figure 2. Distribution of mismatches between the target sequence and identified off-target sequences. Off-targets sequences were extracted from the ITR-Seq results for AAV-M1PCSK9 (at a dose of 3 × 10^13^ or 6 × 10^12^ GC/Kg, panels a and b) and AAV-M2PCSK9 (6 × 10^12^ GC/kg dose, panels c and d) groups at d17. Thirty-one top-ranked (according to the number of ITR-Seq reads) off-target sequences, with a length of 22 bp and with no more than 10 mismatches, were retained for analysis. Location of the off-target sites are shown on the left and mismatches between the off- and on-target sequences are highlighted. The data to generate the WebLogo [43] shown on top were the selected off-target sequences for each group multiplied by the reported number of ITR-Seq reads (Dataset S1). [file 12864_2020_6655_MOESM3_ESM.tif]
