# Supplementary material for: Correction to: ITR-Seq, a next-generation sequencing assay, identifies genome-wide DNA editing sites in vivo following adeno-associated viral vector-mediated genome editing
Source: BMC Genomics. 2020 Nov 20;21:810. doi: 10.1186/s12864-020-07039-2 (PMC7679980; doi:10.1186/s12864-020-07039-2)
Supplement: Supplementary file 3 — Additional file 3: Table S2. ITR-Seq rank of GUIDE-Seq-identified off-target events. [file 12864_2020_7039_MOESM3_ESM.docx]

**S2 Table. ITR-Seq rank of GUIDE-Seq-identified off-target events.**

GUIDE-Seq-identified off-target events, validated by amplicon sequencing analysis on days 17/18 in liver samples obtained from macaques treated with AAV8-M1PCSK9 or AAV8-M2PCSK9 at the indicated doses^24^ and their corresponding ITR-Seq rank. Bold typeface indicates the off-target events in which the reported indel percentage quantified by amplicon sequencing was significantly higher than untreated controls. N.I., not identified by ITR-Seq.

| **Nuclease:** | **AAV8-M1PCSK9** | | | |  | **AAV8-M2PCSK9** | |
| --- | --- | --- | --- | --- | --- | --- | --- |
| **AAV dose:** | 3x10^13^ GC/kg | 6x10^12^ GC/kg | 2x10^12^ GC/kg | 2x10^12^ GC/kg |  | 6x10^12^ GC/kg | 6x10^12^ GC/kg |
|  |  |  |  |  |  |  |  |
| High rank |  |  |  |  | High rank |  |  |
| Chr5:112049529 | **199** | **66** | **30** | **62** | Chr10:72232623 | **9** | **6** |
| Chr20:69811042 | **8** | **2** | **5** | **11** | Chr5:112049529 | **N.I.** | **69** |
| Chr7:123575698 | **N.I.** | N.I. | N.I. | N.I. | Chr19:51609207 | N.I. | N.I. |
| Chr12:10658914 | **157** | **92** | **N.I.** | **N.I.** | Chr19:31971930 | **28** | **14** |
| Chr12:51647755 | **90** | **37** | **36** | **N.I.** | Chr16:48383076 | **6** | **7** |
| Chr13:92389310 | **41** | **58** | **54** | **23** | Chr16:41164165 | N.I. | N.I. |
| Chr16:49265525 | **2** | **5** | **2** | **4** | Chr9:53019653 | **4** | **4** |
| Chr13:43000760 | **6** | **4** | **6** | **12** | Chr14:11716320 | **21** | 13 |
| Chr6:2022570 | **34** | **21** | **22** | **9** | Chr14:69311382 | **10** | **8** |
| Chr5:139700784 | **12** | **N.I.** | 8 | **13** | Chr7:123575698 | N.I. | N.I. |
| Chr9:114398062 | **14** | **11** | **10** | **10** | Chr3:169340141 | N.I. | N.I. |
| Chr9:53019653 | **9** | **17** | **11** | **19** | Chr5:178494103 | N.I. | N.I. |
| Chr10:22429622 | **23** | **16** | **18** | **14** | Chr12:51647755 | **N.I.** | **N.I.** |
| Chr12:46743016 | **19** | **38** | **77** | **N.I.** | Chr16:49265525 | **2** | **5** |
| Chr10:72232623 | N.I. | **28** | **84** | **N.I.** | Chr6:2022570 | **37** | **N. I.** |
| Low rank |  |  |  |  | Low rank |  |  |
| Chr7:167169127 | **1242** | N.I. | N.I. | N.I. | Chr19:5020267 | N.I. | N.I. |
| Chr11:102820504 | **1476** | **N.I.** | **N.I.** | N.I. | Chr3:75646089 | **N.I.** | N.I. |
| Chr1:62000776 | **1289** | N.I. | N.I. | N.I. | Chr14:13573718 | **88** | 38 |
| Chr18:44590418 | N.I. | N.I. | N.I. | N.I. | Chr10:91307567 | **67** | 52 |
| Chr15:80157785 | N.I. | **N.I.** | N.I. | **N.I.** | Chr1:139404583 | N.I. | N.I. |
| Chr7:119527355 | N.I. | N.I. | N.I. | N.I. | Chr12:30957735 | N.I. | N.I. |
