# Supplementary material for: Correction to: ITR-Seq, a next-generation sequencing assay, identifies genome-wide DNA editing sites in vivo following adeno-associated viral vector-mediated genome editing
Source: BMC Genomics. 2020 Nov 20;21:810. doi: 10.1186/s12864-020-07039-2 (PMC7679980; doi:10.1186/s12864-020-07039-2)
Supplement: Supplementary file 5 — Additional file 5: Summary of Corrections. [file 12864_2020_7039_MOESM5_ESM.docx]

**Summary of corrections**

**Supplementary Dataset 1** (filename: 18-14 Dataset S1 BMC Genomics FINAL.xlsx)

- Tab names were corrected as follows

| **Old tab name** | **New tab name** |
| --- | --- |
| AAV8-M1PCSK9 2E12 Animal 1 d17 | AAV8-M2PCSK9 6E12 Animal 2 d18 |
| AAV8-M1PCSK9 2E12 Animal 1 d129 | AAV8-M2PCSK9 6E12 Animal 2 d128 |
| AAV8-M1PCSK9 2E12 Animal 2 d17 | AAV8-M2PCSK9 6E12 Animal 1 d18 |
| AAV8-M1PCSK9 2E12 Animal 2 d129 | AAV8-M2PCSK9 6E12 Animal 1 d128 |
| AAV8-M2PCSK9 6E12 Animal 1 d18 | AAV8-M1PCSK9 2E12 Animal 1 d17 |
| AAV8-M2PCSK9 6E12 Animal 1 d128 | AAV8-M1PCSK9 2E12 Animal 1 d129 |
| AAV8-M2PCSK9 6E12 Animal 2 d18 | AAV8-M1PCSK9 2E12 Animal 2 d17 |
| AAV8-M2PCSK9 6E12 Animal 2 d128 | AAV8-M1PCSK9 2E12 Animal 2 d129 |

**Corrected sentences in article** (corrections highlighted in yellow)

**Results**

We took a liver biopsy of each macaque on days 17/18 and 128/129 post-vector administration to evaluate on- and off-target editing.

The number of off-target sites decreased as a function of time (e.g., day 17/18 had more off-target sites than day 128/129; see Fig. 2a).

We observed a dose-dependent effect among the non-human primates treated with AAV-M1PCSK9, where the highest AAV-M1PCSK9 dose (3x10^13^ GC/kg) resulted in the highest number of off-target sites (2,332 off-target sites at d17) while those administered with the lowest tested dose (2x10^12^ GC/kg) resulted in the lowest number of off-target sites for this group (105 and 194 off-target sites at d17, Fig. 2a).

In total, at day 17 post-AAV administration we observed 1,170 different off-target sites after administering AAV8-M1PCSK9 at a dose of 6x10^12^ GC/kg.

By contrast, at day 18 post-AAV administration we only observed 138 and 120 off-target sites in the two macaques that received AAV8-M2PCSK9 at the same dose (Fig. 2a).

The distribution of ITR-Seq reads for each treated non-human primate at day 17/18 and d128/129 is shown in Figure S1.

Interestingly, we observed an increase in the on-target percentage from d17/d18 to d128/d129, indicating that by day 128/129 between 60 and 90% of the AAV-integrated sequences are in the on-target region (Fig. S1).

This AAV integration frequency was significantly different (Wilcoxon signed-rank test) between the on- and off-target regions (p = 0.031 at day 128/129 post-AAV administration).

We then investigated the presence of ITR sequences in these loci by AMP-Seq, as previously described (26, 32), using gene-specific primers flanking the identified off-target sequences (Table S1).

We found reads containing ITR sequences in 24 (for the 3x10^13^ GC/kg dose) or 21 (for the 6x10^12^ GC/kg dose) out of 27 interrogated loci, with the highest percentage of ITR integration (ITR-containing reads) corresponding to those off-target sites with high ITR-Seq rank (ITR-containing reads (%) column, Table S1).

For some of these loci, we could not detect ITR sequences integrated by the AMP-Seq assay (Table S1)

Similarly, we were able to observe ITR sequences in a couple of sites not shown in the ITR-Seq results (e.g., 20:359062-359285 and 7:165269225-165269449 for liver samples of animals treated with 3x10^13^ GC/kg of AAV8-M1PCSK9; tab AAV8-M1PCSK9 3E13 d17, Dataset S1).

However, we found these sites with the ITR-Seq method in animals treated with 6x10^12^ GC/kg of AAV8-M1PCSK9 (tab AAV8-M1PCSK9 6E12 d17, Dataset S1), suggesting that our current protocol does not capture 100% of the ITR integration sites.

The distribution of matches to the on-target sequence appeared to follow a Gaussian distribution with a mean of 15 - 17 nucleotides.

This indicates that the majority of ITR-Seq-identified off-target sites have five to seven mismatches between the targeted DNA sequence motif and the genomic DNA sequence of each target site (Fig. 2c).

Unlike ITR-Seq-identified sequences, random sequences share an average of 9-10 mismatches with the intended target sequence.

We found that these mismatches were more likely to occur in particular nucleotide positions (nucleotides 4, 12, 13 and 21; Fig. S2), while some nucleotides remained unchanged between the on- and off-target sites (nucleotides 2, 5, 11, 15, 16, and 18; Fig. S2).

By identifying off-target sites with no homology to the indented target sequence, we found approximately the same number of off-target sites across two independent experiments using either M1PCSK9 (1003 and 1375, for GUIDE-Seq experiment 1 and 2, respectively) or M2PCSK9 (506 and 577, for GUIDE-Seq experiment 1 and 2, respectively, Dataset S2).

For this study in rhesus macaques, we performed ITR-Seq on DNA samples from liver biopsies taken on day 17/18 post-nuclease administration.

Most (51.7-82.8%) off-target sites were identified exclusively by ITR-Seq, and not by GUIDE-Seq (see colored sections of Fig. 3).

We counted off-target sites that exhibited a significantly higher indel percentage than untreated peripheral blood mononuclear cell (PBMC) DNA control samples as positive (bold typeface in Table S2).

Among macaques administered with 3x10^13^ and 6x10^12^ GC/kg of AAV8-M1PCSK9, ITR-Seq only failed to identify two high-rank and two low-rank positive GUIDE-Seq off-target sites (N.I. in bold typeface in Table S2).

Among macaques administered with AAV8-M2PCSK9, ITR-Seq correctly identified most of the positive off-target sites and only missed two high- and one low-rank positive off-target sites in one animal and two high-rank positive off-target sites in the other macaque (Table S2).

Body of Table 1 – ‘Target locus’ column - Ass1

Aside from one animal treated with AsCpf1-sgRNA2, the on-target locus (mAss1) was the target with the highest number of reads in ITR-Seq analysis.

Importantly, mice treated with AAV8-SaCas9 exhibited the highest frequency of on-target ITR integration events across all treated samples (Table S3).

Despite showing comparable on-target editing, we observed lower on-target ITR integration events in livers treated with AAV8-LbCpf1 versus AAV8-SaCas9 (Table S3).

All of the ITR-Seq-identified off-target sites for the CRISPR nucleases resided within annotated mouse genes, with the most common site being the on-target locus (Table S3).

One off-target site that we identified for the AAV8-SaCas9 sgRNA was located within the locus of a known oncogene, NOTCH2 (Table S3).

Thus, only a higher dose of AAV8-sgRNA1 led to editing at this low abundance off-target site (Table S3).

**Discussion**

Analysis of non-human primate samples by ITR-Seq showed a clear decrease in the number of off-target sites, as well as the total number of ITR-Seq reads for the off-target sequences from d17/d18 to d128/d129 (Fig. 2a).

In our studies in mice, the most common nuclease-independent ITR integration events occurred in the Gm10800 and albumin genes (Table S3).

**Methods**

Liver biopsies were performed at 17 and 129 days (for AAV8-M1PCSK9) or 18 and 128 days (for AAV8-M2PCSK9) post-vector administration (26).

Additional newborn mice (n=2 per group) were co-administered with vector expressing SaCas9 or LbCpf1 as described above at a dose of 10^11^ or 3x10^11^ GC/mouse, with the second vector expressing Ass1-specific-sgRNA and the hFIX transgene (AAV8.U6.sgRNA.mASS1.TBG.hFIX) at a dose of 10^12^ GC/mouse.

**Availability of data and materials**

Datasets S1 and S2 are available at BioProject (accession number PRJNA609560).

**References**

References (please delete any extraneous DOIs; lines 675, 676, and 677)

**Figure 1 legend**

**b.** Secondary structure of the AAV2 5' ITR (NC_001401.2).

**Figure 2 legend**

Samples were collected on day 17/18 and 128/129 following vector administration.

**Supplemental Figure 1 legend**

Analysis was performed on the ITR-Seq results for liver biopsies at d17/d18 and d128/d129 from non-human primates treated with the indicated nuclease and AAV dose.

**Supplemental figure 2 legend**

Off-targets sequences were extracted from the ITR-Seq results for AAV-M1PCSK9 (at a dose of 3x10^13^ or 6x10^12^ GC/kg, panels a and b) and AAV-M2PCSK9 (6x10^12^ GC/kg dose, panels c and d) groups at d17/d18.
